# Depth diversity gradients of macrophytes: Shape, drivers, and recent shifts

**DOI:** 10.1002/ece3.8089

**Published:** 2021-09-23

**Authors:** Anne Lewerentz, Markus Hoffmann, Juliano Sarmento Cabral

**Affiliations:** ^1^ Ecosystem Modelling Center for Computational and Theoretical Ecology (CCTB) University of Würzburg Würzburg Germany; ^2^ Limnological Station Chair of Aquatic Systems Biology Technical University of Munich Munich Germany

**Keywords:** aquatic plants, biodiversity gradients, biodiversity hypotheses, deep lakes, Germany, Water Framework Directive

## Abstract

Investigating diversity gradients helps to understand biodiversity drivers and threats. However, one diversity gradient is rarely assessed, namely how plant species distribute along the depth gradient of lakes. Here, we provide the first comprehensive characterization of depth diversity gradient (DDG) of alpha, beta, and gamma species richness of submerged macrophytes across multiple lakes. We characterize the DDG for additive richness components (alpha, beta, gamma), assess environmental drivers, and address temporal change over recent years. We take advantage of yet the largest dataset of macrophyte occurrence along lake depth (274 depth transects across 28 deep lakes) as well as of physiochemical measurements (12 deep lakes from 2006 to 2017 across Bavaria), provided publicly online by the Bavarian State Office for the Environment. We found a high variability in DDG shapes across the study lakes. The DDGs for alpha and gamma richness are predominantly hump‐shaped, while beta richness shows a decreasing DDG. Generalized additive mixed‐effect models indicate that the depth of the maximum richness (*D*_max_) is influenced by light quality, light quantity, and layering depth, whereas the respective maximum alpha richness within the depth gradient (*R*_max_) is significantly influenced by lake area only. Most observed DDGs seem generally stable over recent years. However, for single lakes we found significant linear trends for *R*_max_ and *D*_max_ going into different directions. The observed hump‐shaped DDGs agree with three competing hypotheses: the mid‐domain effect, the mean–disturbance hypothesis, and the mean–productivity hypothesis. The DDG amplitude seems driven by lake area (thus following known species–area relationships), whereas skewness depends on physiochemical factors, mainly water transparency and layering depth. Our results provide insights for conservation strategies and for mechanistic frameworks to disentangle competing explanatory hypotheses for the DDG.

## Graphical abstract

The first in‐depth characterization of depth diversity gradients (DDG) of submerged macrophytes across different lakes shows a hump‐shaped curve with depth for alpha and gamma richness and decreasing curve for beta richness. The DDG amplitude seems driven by lake area (thus following known species–area relationships), whereas skewness depended on physiochemical factors, mainly water transparency and layering depth. Most observed DDGs seem generally stable over recent years, but significant trends are found for some richness components and lakes.

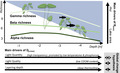

## 1 INTRODUCTION

Describing and explaining biodiversity gradients have been central goals of biogeography and ecology since the beginning of the respective fields (Gaston, 2000). Improving our understanding of the biodiversity gradients and their drivers is still an important requirement to deal with impending species loss. Therefore, many studies have explored environmental gradients as explanatory variables for biodiversity patterns along different geographic scales (Rahbek, 2004; Whittaker et al., 2007) such as (a) latitude (Stehli et al., 1969; Rohde, 1992; Pontarp et al., 2019; Etienne et al., 2019), (b) elevation (Colwell & Rangel, 2010; Graham et al., 2014; Hutchinson, 1953; Lomolino, 2001; Nogués‐Bravo et al., 2008; Rahbek, 1995; Rahbek et al., 2019; Sanders & Rahbek, 2012), (c) tree height in forests (Petter et al., 2016), (d) depth in soils (Jakšová et al., 2019; Rendoš et al., 2016), or (e) depth in water (Gong et al., 2015; Rex & Etter, 1998; Smith & Brown, 2002). These geographic gradients share some environmental gradients, which are expected to influence spatial structuring of diversity gradients, for example, temperature, light, or seasonality. However, the shorter the spatiotemporal scales are, the less confounding biogeographical contingencies there are, such as the legacy of the glacial cycles on latitudinal gradients, and dispersal/connectivity limitations. Hence, studying gradients expressed at short spatiotemporal extents may provide valuable insights on drivers of biodiversity. Still, the short spatiotemporal gradients, like depth in freshwaters, are often overlooked.

Freshwater ecosystems have a high biodiversity with a high rate of species loss (Gatti, 2016; He et al., 2017; Strayer & Dudgeon, 2010), exceeding those of terrestrial systems (Dudgeon et al., 2006). Nonetheless, studies that focus on the short spatiotemporal diversity gradient in freshwater are surprisingly scarce, although light gradients in freshwater must represent a very strong driver. The few existing studies seem to show predominantly a general decrease of biodiversity along the depth gradient, for example, for bacteria (Cantonati et al., 2014; Zhao et al., 2019), chironomids (Zhao et al., 2019) or diatoms (Kingsbury et al., 2012; Stoof‐Leichsenring et al., 2020), or hump‐shaped patterns along depth, for example, for diatoms (Zhao et al., 2019) or submerged macrophytes (Ye et al., 2018). Macrophytes are, however, comparatively less studied.

Macrophytes play a pivotal role in lakes by reducing nutrient concentrations (Song et al., 2019), by providing food for a lot of other species (Bakker et al., 2016), and by giving shelter to a large number of other aquatic organisms like zooplankton, juvenile fish, and amphibians (Jeppesen et al., 1998). However, there are several knowledge gaps on macroecology of freshwater plants (Alahuhta et al., 2020). Some aspects of depth gradients in macrophytes can also be found in the literature, with previous studies mainly focus on depth limits of species (Domin et al., 2004; Middelboe & Markager, 1997; Søndergaard et al., 2013), growth of single species dependent on depth (Fu et al., 2018; Li et al., 2020; Xu et al., 2020), functional diversity along depth (Fu et al., 2017), or biomass of macrophytes along depths (Chambers & Kaiff, 1985; Collins et al., 1987). However, the depth pattern of submerged macrophytes species richness is sparsely studied (Bolpagni et al., 2016; Fu et al., 2015; Fu, Zhong, Yuan, Ni, et al., 2014; Fu, Zhong, Yuan, Xie, et al., 2014; Ye et al., 2018). The few studies that have looked at depth distribution of macrophytes in lakes mainly focused on Lake Erhai in Yunnan Province, China. They report a hump‐shaped pattern along the water depth gradient for species richness and community biomass of submerged macrophyte species (Ye et al., 2018). Looking at all functional types including emergent species, Lake Erhai shows a significant decrease in taxonomic and functional diversity along the water depth gradient and its niche differentiation (Fu et al., 2014b, 2015; Fu, Zhong, Yuan, Xie, et al., 2014). Also, hump‐shaped and decreasing patterns of species numbers along depth were found in four Italian lakes, changing patterns with time (Bolpagni et al., 2016). Still, it remains unclear if the described pattern of macrophytes is generalizable across multiple lakes and whether it stays robust over time.

The lack of macrophyte diversity gradient studies is intriguing because the environmental gradients along lake depth represent one of the sharpest found in nature, with strong variation over just few meters. With increasing lake depth, multiple abiotic factors that influence the growth of macrophytes (light, temperature, nutrients, water quality, disturbances/hydrologic variability) drastically change (Bornette & Puijalon, 2011). Light is gradually attenuated with increasing depth due to absorption and scattering, resulting in a specific reduction of light quality and quantity depending on depth and on the water turbidity. Water temperature in deep lakes does not decrease gradually, but rather abruptly with depth (Bornette & Puijalon, 2011). The formation of thermally stratified lakes results in an abrupt thermocline, especially during growing season. The thermocline influences the within‐lake fluid dynamics in each thermal layer, further leading to stratified gradients in nutrients and water quality components during stratification (Bornette & Puijalon, 2011). Moreover, mechanical disturbances, like wind or waves, lose their influence gradually with depth (Van Zuidam & Peeters, 2015). The probability that water‐level fluctuations result in drying up the soil (Evtimova & Donohue, 2016) is also reduced, and browsing pressure by waterfowl decreases with depth (Bakker et al., 2016). How these different environmental gradients influence the species richness of macrophytes remains unclear, although knowing the processes shaping species diversity might help to predict how global change will affect biodiversity and how management strategies might mitigate potential negative diversity responses.

This study aims to describe the depth distribution of macrophyte diversity, to assess the relative importance of its drivers, and to search for recent shifts. Specifically, we ask the following questions:

**Table:** 

1.	1.1. What is the general shape of the depth diversity gradient (DDG) of submerged macrophytes in deep lakes?
	1.2. Are there differences between lakes and diversity components (alpha‐, beta, gamma richness)?
2.	What are the drivers for macrophyte DDG?
3.	3.1. Has the DDG been stable over recent years?
	3.2. Are temporal trends general or lake‐specific?

To address these questions, we use macrophytes occurrence data of 274 transects taken over 13 years across 28 natural deep lakes in Bavaria that were mapped for monitoring in relation to the European Water Framework Directive. We expect a hump‐shaped DDG (question 1.1) corresponding to previous scattered empirical evidence and following the typical patterns found along elevation (Nogués‐Bravo et al., 2008). We assume no strong differences between lakes and diversity components (question 1.2), as the pattern along elevation is shown to be generalizable for alpha and gamma richness (Bhatta et al., 2018) and as we consider beta richness as additive partitioning of alpha and gamma richness. To tackle question 2, we test whether the shape of the DDG can be explained by geographic and physical–chemical characteristics of the lakes. We expect water quality to have a high degree of influence, since water quality affects resource availability (light, temperature) (Bornette & Puijalon, 2011). Finally, we assess, with regard to questions 3.1–3.2, whether there have been detectable temporal changes in the DDG. We suppose that the DDG is a quite stable pattern over time, as macrophytes react slowly to changes (Bakker et al., 2013). However, due to the overall warming in annual average water temperature during the last decades we expect that species richness increases, as invasive species are expected, and warm‐adapted species might expand. Our results provide the most refined and extensive assessment of macrophyte biodiversity patterns in freshwater lakes up to date, giving insights for the development of long‐term conservation strategies for freshwater systems in general.

## 2 MATERIALS AND METHODS

### 2.1 Data and study area

Bavaria has a wide variety of lakes, which vary in size, depth, altitude, and physiochemical parameters. Information about lake surface area, maximal lake depth, and mixing regime was provided by Bayerisches Landesamt für Wasserwirtschaft (1987). We obtained data for water‐level statistics and physical–chemical data from the hydrological service of Bavaria (https://www.gkd.bayern.de/). We selected the physical–chemical parameters that were measured for the largest number of lakes: *chloride* (*Chl*), *conductivity* (*Cond*), *total nitrogen* (*N_tot_*), *ammonium* (*NH_4_^+^*), *nitr* *ate* (*NO_3_^−^*), *dissolved oxygen* (*O_2diss_*), *total phosphorus* (*P_tot_*), *pH*, *silicate* (*SiO_2_*), *water temperature* (*Temp*), *transparency* (*Transp*), and *spectral absorption coefficient at 254 nm* (*SAC*). Although some of the parameters seem redundant at first glance, all parameters are also indicators for environmental conditions with secondary or indirect effects on the macrophytes. For example, both the concentration of *total nitrogen* and the concentration of *nitrate* or even *a* *mmonium* can influence biodiversity. The *total nitrogen* indicates the basic nutrient situation, while the *ammonium* or *nitrate* concentrations can affect species with certain nitrogen preferences (Nelson et al., 1981). We did not include *Chl‐a* measurements as they were not consistently available and *phosphorus* concentration is supposed to be the better predictor for trophic state and lake productivity in central European lakes. Physical–chemical data include monthly measurements at the deepest point of the lake.

**FIGURE 1:**
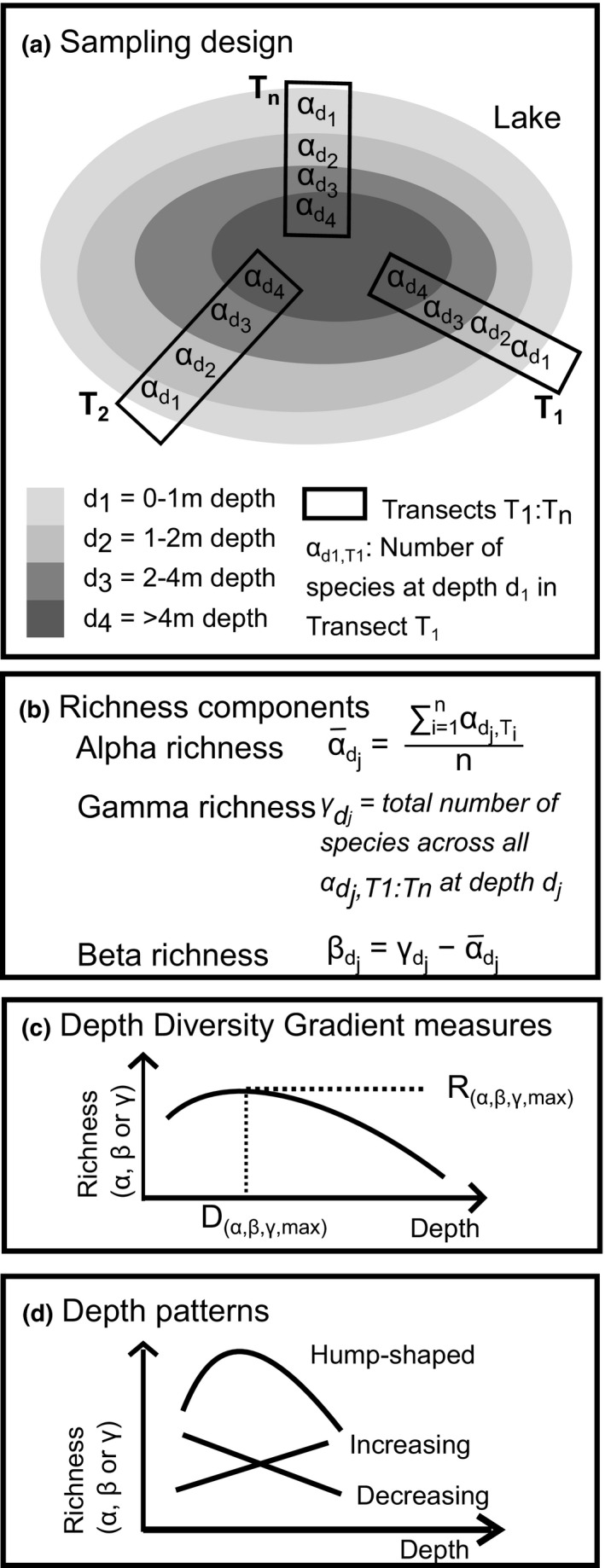
Experimental design and analyzed variables. (a) Sampling design of empirical data: Each lake was mapped along representative number of transects in four different depths. Three species richness components were calculated (b) alpha richness, gamma richness, and beta richness. For all three depth curves, richness peaks (c) is characterized by *D*_*max*_ and *R*_*max*_ as depth diversity gradient measures. Resulting depth pattern types (d) can be decreasing, hump‐shaped, and increasing

Macrophyte data were also extracted from the Bavarian State Office for the Environment (www.lfu.bayern.de). The macrophyte data were recorded for the EU‐Water Framework Directive Monitoring following an official sampling strategy (Schaumburg et al., 2015) and include vegetation surveys for all large lakes of Bavaria (>0.5 km² surface area) for at least one and maximum five different years. At each lake, macrophyte data for several transects perpendicular to the shoreline at characteristic sections are available (see sampling strategy in Figure 1a). Each transect is considered from shoreline to the lowest macrophyte occurrence and is subdivided along the depth gradient into four depths (0 to −1 m; −1 to −2 m; −2 to −4 m; >−4 m). At each depth, the frequency of all species is sampled in five steps following the scale of Kohler (1978), an estimate of abundance.

### 2.2 Data preparation

Data preparation and analyses were done in R 3.5.3 (R Core Team, 2019). The preprocessed data and the code for the data analysis is provided as Research Compendium on github (https://github.com/AnneLew/Lewerentz‐etal_2021_Macrophytes‐DDG).

To have comparable conditions, we selected all lakes from the dataset that are deep (maximum depth >10 m), not artificial, dimictic, and have a natural water‐level dynamic (i.e., not influenced by storage power plants) and at least one sampling repetition. More information about the single selected lakes and their morphometry can be found in the Supplementary material.

To describe the local *water‐level fluctuation* (*WLF*), we calculated for each lake the difference between mean high water (*MHW*) and mean low water (*MLW*).

WLF=MHW‐MLW

Based on the monthly abiotic measurements, we calculated annual means for all chemical–physical variables based on monthly measurements at the lake surface. For this calculation, we considered measurement campaigns with at least eight monthly values available. Values below the detection were assumed to be zero. To describe the water layering of the lakes, we used the standard deviation of the water temperature measurements of surface, −2, −4, and −6 m depth (*Tempsd*). The available geographic and chemical–physical variables and their mean, standard deviation, median, and minimal and maximal values are given in Table 1.

**TABLE 1:** Overview of the number of datapoints (N), minimum values (Min), maximum values (Max), mean, standard deviation (SD), and median of the abiotic data (geographic variables: fixed values per lake; physical–chemical variables: changing over years, annual means were used) used as indicators in the studied lakes

	Variable (unit)	Abbrev.	*N*	Min (annual mean)	Max (annual mean)	Mean (annual mean)	SD (annual mean)	Median (annual mean)
Geographic variables	Lake area (ha)	*Area*	12	135.00	7,990.00	1,840.00	2,654.00	624.50
	Water‐level fluctuation (m)	*WLF*	12	0.20	1.18	0.58	0.28	0.59
Physical–chemical variables	Chloride (mg/l)	*Chl*	27	0.33	14.98	7.58	3.94	7.61
	Conductivity (µS/cm)	*Cond*	27	158.55	372.44	292.57	61.96	309.20
	Total Nitrogen (mg/l)	*N_tot_*	27	0.19	1.22	0.66	0.22	0.65
	Ammonium (mg/l)	*NH_4_^+^*	27	0.00	0.09	0.02	0.02	0.01
	Nitrate (mg/l)	*NO_3_^−^*	27	0.00	1.09	0.37	0.28	0.30
	Dissolved Oxygen (mg/l)	*O_2_* *_diss_*	27	9.16	11.41	10.23	0.51	10.39
	Total Phosphorus (mg/l)	*P_tot_*	27	0.00	0.02	0.01	0.00	0.01
	pH (‐)	*pH*	27	8.04	8.49	8.32	0.11	8.35
	Silicate (mg/l)	*SiO_2_*	27	0.00	2.86	1.68	0.88	1.64
	Water temperature (°C)	*Temp*	27	9.64	19.24	13.38	2.09	13.32
	Transparency (cm^−1^)	*Transp*	27	242.50	1,197.27	451.74	198.39	410.00
	Spectral absorption coefficient at 254 nm (1/m)	*SAC*	27	0.00	24.83	8.85	7.84	5.35
	Standard deviation of temperature measured at 0 m, −2 m, −4 m, and −6 m depth (°C)	*Tempsd*	27	0.23	2.24	0.91	0.58	0.62

From the macrophytes surveys, we excluded datasets with (I) just one plot or transect for a lake and year, (II) species that were identified as emergent or floating‐leaved plants, and (III) plants that were not identified down to the species level. Thus, free‐floating submerged plants and rooted submerged plants are considered, as well as submerged forms of emergent or floating‐leaved plants. For further calculations, we transformed the depth ranges to decimal numbers by the mean of their limits. That is, the depth range of 0–1 m was converted to −0.5 m depth, the range of 1–2 m in −1.5 m, 2–4 m in −3.0 m, and >4 m in a depth of −5.0 m.

### 2.3 Species richness components and depth diversity gradient measures

As depth‐independent component of species richness, we calculated gamma richness as the total number of species per lake and year.

As depth‐dependent component of species richness, we determined for every lake and year an additive alpha richness as the number of species per depth range averaged across transects (Figure 1b). The gamma richness for every lake and year was defined as the total number of species per depth range (Figure 1c). We then calculated an additive beta richness as gamma richness minus alpha richness (Tuomisto, 2010) (Figure 1d).

To further characterize the diversity depth gradient, we identified the peak of the richness depth curve (Figure 1c). For each transect, we filtered the depth with the maximal species number. Thereafter, we averaged this valued across transects per lake and year, from now on termed the depth with maximal alpha richness (*D*_(α,max)_). The corresponding maximal species number averaged across transects is termed the maximal alpha richness (*R*_(α,max)_). Similarly, the depth with maximal gamma richness (*D*_(γ,max)_) is the depth of the maximal gamma richness (*R*_(γ,max)_) along depth, and maximal beta depth (*D*_(β,max)_) describes maximal beta richness (*R*_(β,max)_) along depth.

### 2.4 Statistical analysis

We addressed the study questions with several analyses, focusing on different dataset levels dependent on data availability. The *biodiversity dataset* contains all macrophyte recordings (274 mapped transects in 100 field campaigns, mapping of lake in one year is called *field campaign*) of the selected 28 lakes. As no complete information is available for all mapped lakes and years, we compiled two subsets of the *biodiversity dataset*: The *environmental & biodiversity dataset* is a subset dataset with all macrophyte recordings for which all abiotic data (see Table 1) were available. This dataset includes data from 12 lakes, 27 field campaigns, and 147 transects. For the *biodiversity time series dataset*, we selected all lakes for which repeated mappings for at least 3 years were available. This condition was fulfilled for 17 lakes mapped in 73 field campaigns along 194 transects. Analyses for each research question are described below.

For the first question, concerning the general depth distribution pattern, we used the richness components including the different DDG measures and determined pattern types. We plotted as general DDG curves the mean and standard deviation of alpha, beta, and gamma richness for each depth (Question 1.1). We performed simultaneous tests for linear models with multiple comparisons of means using Tukey contrasts that are robust under non‐normality, heteroscedasticity, and variable sample size (Herberich et al., 2010) to compare the richness across depth for significant difference. Furthermore, we plotted the different DDG peaks (DDG measures) for alpha, beta, and gamma richness and determined the corresponding regression line by fitting a linear model. We classified the DDG curves for all three richness measures in four pattern types depending on the depth of the richness curve maximum: decreasing (*D*_max_ > −1m), shallow hump‐shaped (*D*_max_ between −1 and −2 m), deep hump‐shaped (*D*_max_ between −2 and −4 m) and increasing (*D*_max_ < −4 m) (Figure 1d). To determine the correlations between the different diversity components (Question 1.2), we performed a Pearson correlation test between depth‐dependent richness components. Furthermore, we tested for correlations between DDG measures across the different richness components. A chi‐square test helped to look at associations between pattern types and biodiversity components.

For the second question, concerning the drivers of the diversity depth gradient, we analyzed the influence of abiotic data on the DDG using the *environmental & biodiversity dataset*. We log‐transformed the abiotic and biotic data. To show that the diversity metrics of the *environmental & biodiversity dataset* are representative for the diversity metrics of *biodiversity dataset*, we applied the PERMANOVA test *adonis2*, using the R package “vegan” which compares centroids and the variance (Oksanen et al., 2019). A nonsignificant result (*p* > .05) confirms that centroids and variance of two groups are not different (Supporting information). To identify the driving factors on the richness peaks, we used generalized additive mixed‐effect models (GAMMs), computed with the R package “gamm4” (Wood, 2011). *D*_(α,β,γ,max)_ and *R*_(α,β,γ,max)_ were used as response variables, the lake as random effect. To reduce the high correlations between abiotic factors (Pearson correlation test), we performed a principal component analysis (PCA) and named the main axis (>80% variance) after the corresponding abiotic factor, whenever an axis encompassed more than 40% of the variation of a variable. We used the loadings of the main PCA axes (>80% variance) as explanatory variables for the GAMM. We constructed a full model with all PCA axes; then, we stepwise excluded the least significant terms until obtaining a minimal model (Wood, 2008).

To answer questions 3.1 and 3.2, concerning the temporal change of the depth diversity gradient, we used the *biodiversity time series dataset*. First, we calculated the invariability coefficient (IC) as inverse of the coefficient of variation (CV):

$$\text{IC}=\frac{1}{\text{CV}}=\frac{1}{\text{sd}/\text{mean}}=\frac{\text{mean}}{\text{sd}}$$

The IC is a statistical tool to evaluate the degree of invariability also for datasets with different means (Question 3.1). To check for temporal trends, we built simple linear regression models for depth‐independent gamma richness and the DDG measures, *D*_(α,β,γ,max)_ and *R*_(α,β,γ,max)_, as response variables and time as explanatory variable for (a) the complete dataset and (b) each individual lake. We identified all models that showed significant linear trends (*p* < .1) and characterized the direction of their slopes (Question 3.2).

## 3 RESULTS

For the dataset of all macrophyte recordings (*biodiversity dataset*), a total of 75 submerged species is documented in Bavaria. The available taxonomic groups are mainly Spermatophytes (51 species), Charophytes (20 species), Bryophytes (two species), and Pteridophytes (two species). The complete abiotic and biotic data (*environmental and biodiversity dataset*) cover 57 different species, whereas the *biodiversity time series dataset* included 66 species. The total (depth‐independent) gamma richness per lake ranges from 5 to 34 species of submerged macrophytes. The mean gamma richness averaged across lakes is 15.36 species with a standard deviation of 6.27 species.

### 3.1 The depth diversity gradient (DDG) patterns of macrophytes

**FIGURE 2:**
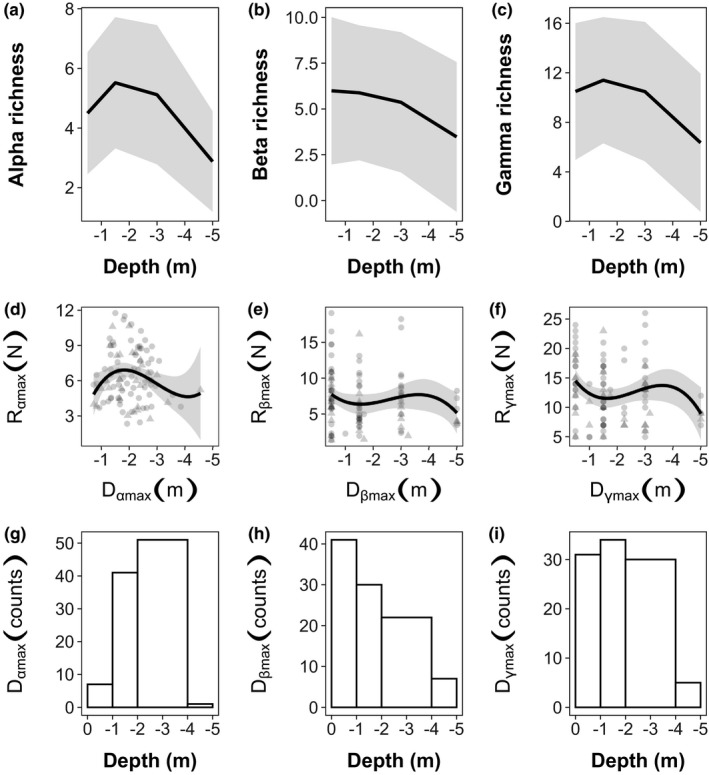
Depth diversity gradient (richness along depth) total mean (black line) and *standard deviation* (gray area) for alpha (a), beta (b), and gamma richness (c). The individual depth curves of alpha richness for all lakes and years are shown in the Supporting information. Points show the individual DDG peaks (DDG measures) of all field campaigns (d–f). Point shapes indicate different dataset levels: round and triangular points = full dataset with all biotic data; triangular points = nested subset, for which each biotic datapoint has a corresponding abiotic datapoint. Intermediate values between depth classes result as mean from lakes with equal species numbers in two different depths. Histograms (g–i) show distribution of *D*_*max*_, which can be interpreted as distribution of pattern types (see definition in Figure 1)

The mean depth pattern of submerged macrophytes' alpha and gamma richness is hump‐shaped, showing a peak between −1 and −2 m, respectively (Figure 2a,c). The mean alpha richness at the hump's peak is 4.5 species (*SD* = 2.2), whereas the mean gamma richness peak is 11.4 species (*SD* = 5.1). In contrast, beta richness shows a decreasing curve with its highest richness being 7.0 species (*SD* = 4.0) between surface and −1 m depth (Figure 2b). However, all three richness components show high standard deviations in the depth classes. They vary across depth classes between 1.9 and 2.3 species for alpha richness, between 3.7 and 4.1 for beta richness and from 5.1 till 5.6 for gamma richness (see individual DDG curves for all lakes in Supporting information).

Comparing the richness components across depths revealed only significant differences between mid‐ and greater depths, but not for shallower depth (Supporting information).

Plotting the DDG measures for all three richness components (Figure 2d–f), we find a hump‐shaped pattern for alpha richness and a bimodal pattern for beta and gamma richness.

Looking at the DDG patterns of single field campaigns, for alpha richness, hump‐shaped curves with a peak between −2 and −4 m are most frequent (52%) (Figure 2g–i). For beta richness, the majority are decreasing curves (40%), while hump‐shaped curves with a peak between −1 and −2 m were slightly prevailing (39%) for gamma richness. All depth pattern types are found for all three measures.

The three depth‐dependent richness measures are significantly correlated with one another (*p* < .05). Beta and gamma richness (cor = 0.95) show the strongest correlation, followed by alpha and gamma richness (cor = 0.85) and alpha and beta richness (cor = 0.64). *D*_max_ and *R*_max_ do not correlate within the respective richness components (*p* < .05). However, the *D*_max_ values across the three richness components correlate with each other. Similarly, *R*_max_ also correlates across the three richness components (*p* < .05). For correlation coefficients see Supporting information. However, a chi‐square test shows that the association between pattern types and richness components is statistically significant (*p* = .0005).

### 3.2 Drivers of the depth diversity gradients

The DDG measures correlate with some of the abiotic variables (Supporting information). *R*_(α,max)_ correlates highly significantly (*p* < .01) with *area* (cor = 0.53, *p* < .01), *WLF* (cor = 0.54, *p* < 0.01), *Cond* (cor = 0.53, *p* < .01), *NH_4_^+^* (cor = −0.5, *p* < .01), *SiO_2_* (cor = 0.62, *p* < .01), and *SAC* (cor = 0.6, *p* < .01). *R*_(β,max)_ correlates highly significantly (*p* < .01) with *area* (cor = 0.55, *p* < .01). *R*_(γ,max)_ correlates highly significantly (*p* < .01) with *area* (cor = 0.57, *p* < .01) and *WLF* (cor = 0.56, *p* < .01). *D*_(α,max)_ correlates highly significantly (*p* < 0.01) with *O_2__diss_* (cor = −0.54, *p* < .01), *P_tot_* (cor = 0.6, *p* < .001), *Transp* (cor = −0.67, *p* < .001), and *Tempsd* (cor = 0.59, *p* < .01). *D*_(β,max)_ and *D*_(γ,max)_ do not correlate highly significantly (*p* < .01) with any of the abiotic variables.

Abiotic and biotic variables are correlated with one another in a complex fashion (Supporting information). Strongest positive correlations (cor > 0.7 or <−0.7) within abiotic data were found between *N_tot_* and *NO_3_^‐^* (cor = 0.92, *p* < .01), *Cond* and *SiO_2_* (cor = 0.83, *p* < .01), *Chl*, and *Cond* (cor = 0.73, *p* < .01). Strongest negative correlations showed *Transp* and *P_tot_* (cor = −0.72, *p* < .01) and *Transp* and *Cond* (cor = −0.71, *p* < .01).

**FIGURE 3:**
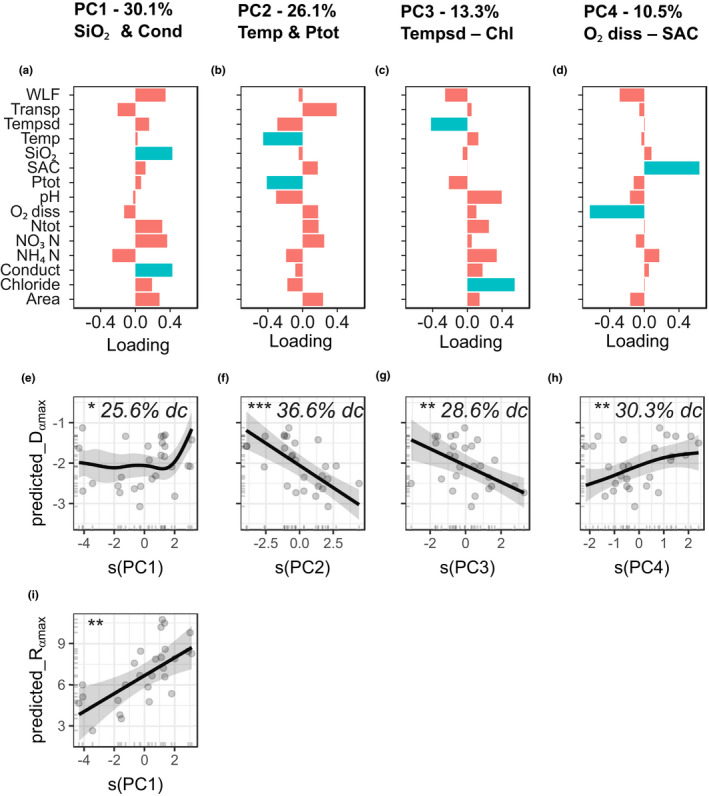
The PCA loadings (a‐d) of the four first axes (81% variation) of a PCA for all abiotic variables were used as explanatory variables of the GAMM. The names of the axes are given by the variables with >40% of the loading (highlighted in blue). The full variable names are given in Table 1. Panels a–d are ordered corresponding to the order of a–d. PCA biplot can be found in the Supporting information. Minimal best models for generalized additive mixed models explaining *D* *_(α,max)_* by PC2 (e), PC4 (f), PC3 (g), and PC1 (h) and *R* *_(α,max)_* by PC1 (i). GAMMs without significant explanatory variables are not shown (*R*_*(β,max)*_, *R*_*(γ,max)*_, *D*_*(β,max)*_, *D*_*(γ,max)*_). Panels e–h are ordered by decreasing drop contribution (dc). The relative contribution of each variable to the full model (% drop contribution) is measured as the drop in deviance explained by the model when the predictor is removed. Significance levels of p‐values: *p < .05; **p < .01; ***p < .001. *R* ^2^_adj_ for *D*_*(α,max)*_ is 73.5%, for *R*_*(α,max)*_ 43.9%

Due to the high correlation coefficients between abiotic factors, we performed a PCA (Supporting information). We use the first four axes (81% of total variation – Figure 3f–i) to address the DDG drivers. The first axis, PC1, can be characterized as the *“SiO_2_ & Conductivity axis”* (both positive with the axis), explaining 30.1% of the variance. The PC2, the second axis, can be described as the “*Temperature & P_tot_ axis*,” as both abiotic variables have the highest (negative) impact (26.1% of variance). The third axis, PC3, can be named the “*Temperature sd – Chloride axis*” (13.3% of variance) as it ranges from most negative variable *Tempsd* to most positive variable *Chl*, while the fourth axis, PC4, shows the “*O_2diss_ – SAC axis*” (10.5% of variance) spanned between *O_2diss_* (most negative) and *SAC* (most positive).

The GAMM showed that *D*_(α,max)_ (*R* ^2^ = 0.73) significantly varies with all four PCA axes (Figure 3a–d). *D*_(α,max)_ decreases with PC2 (*Temperature & P_tot_ axis*) and PC3 (*Tempsd—Chloride axis*) axes, slightly increases with PC4 axis (*O_2diss_—SAC axis*) and increases only for extreme positive values of PC1 axis (*SiO_2_ & Conductivity axis*). The *R*_(α,max)_ (*R* ^2^ = 0.44) is only influenced by the PC1 axis (*SiO_2_ & Conductivity axis*) with a positive linear relationship (Figure 3e). The GAMM analysis for *D*_(β,max),_ *R*_(β,max),_ *D*_(γ,max),_ and *R*_(γ,max)_ had all *R* ^2^ < 2.1% (see results in Supporting information).

### 3.3 Temporal dynamics of the depth diversity gradients

The DDG measures of the different richness components show different degrees of invariability coefficient (*IC*) as measure for stability. The *IC* of Gamma richness (mean = 7.14, *SD* = 3.69), of *D*_(α,max)_ (mean = 6.62, *SD* = 4.11), of *R*_(α,max)_ (mean = 6.36, *SD* = 2.87), of *R*_(β,max)_ (mean = 5.89, *SD* = 2.06), and of *R*_(γ,max)_ (mean = 6.52, *SD* = 2.97) were high in comparison with the *IV* of *D*_(β,max)_ (mean = 1.56, *SD* = 0.48) and of *D*_(γ,max)_ (mean = 1.78, *SD* = 0.53).

For all lakes, depth‐independent gamma richness showed a small but significant trend (*p* < .1) toward more species (Supporting information). Analyzing all lakes together, the DDG measures revealed no significant common trend (*p* > .05) (Supporting information). For individual lakes, slopes of significant linear models of the *D*_max_ and *R*_max_ over years showed mostly positive trends (richness increasing and peaking at shallower depths, see Supporting information). The *D*_(α,max)_ shows two significant positive trends (peak shifting toward water surface—lake Starnberg and lake Tegernsee) and two significant negative trends (peak shifting to deeper waters—lakes Großer Alpsee and Woerthsee). The *D*_(β,max)_ increases significantly at Lake Riegsee, while *D*_(γ,max)_ increases for lakes Riegsee, Staffelsee Nord, and Tegernsee. For total gamma richness, lakes Chiemsee, Staffelsee Nord, Staffelsee Süd, and lake Starnberg show positive trends (Table 2).

**TABLE 2:** Linear model results of the time series analysis of DDG variables (*D*_*max*_ and *R*_*max*_) across richness components for each lake

Lake	Gamma richness	*D*_(α,max)_	*R*_(α,max)_	*D*_(β,max)_	*R*_(β,max)_	*D*_(γ,max)_	*R*_(γ,max)_
Lake Abtsdorf	−	−	−	−	+	−	−
Ammersee	−	+	−	+	−	+	−
Chiemsee	+**	+	−	−	+	+	**+.**
Gr. Alpsee	+	−*	+	−	−	−	+
Grosser Ostersee	−	+	−	+	−	+	−
Hopfensee	+	−	+		+*		**+.**
Lake Niedersonthofen	−	+	−	+	+	+	−
Lake Pelham	+	−	+	+	−	−	+
Riegsee	+	+	+	+*	**+.**	**+.**	+
Schliersee	−	+	−	+	−	−	−
Simssee	+	+	+	+	+	+	+
Staffelsee – Nord	+*	+	+	+	**+.**	**+.**	+
Staffelsee – Sued	+*	+	**+.**	−	**+.**	+	**+.**
Lake Starnberg	**+.**	**+.**	+***	+	+*	+	+***
Tegernsee	+	+*	+	+	+	**+.**	+
Lake Waging	+	−	+	+	+	+	+
Woerthsee	−	−*	+	+	+	+	+
Sign (*p* < .1) pos. slope (*N* lakes)	**4**	**2**	**2**	**1**	**5**	**3**	**4**
Sign (*p* < .1) neg. slope (*N* lakes)	**0**	**2**	**0**	**0**	**0**	**0**	**0**

Note

Bold formated cells indicate a significant trend. Here, +: positive slope of linear model (meaning for *D*_max_: becomes shallower; meaning for *R*_max_: more species); −: negative slope of linear model (meaning for *D*_max_: becomes deeper; meaning for *R*_max_: less species); significance levels of *p*‐values: 0 “***” .001 “**” .01 “*” .05 “.” .1.

## 4 DISCUSSION

### 4.1 The DDG patterns of macrophytes

We showed that submerged macrophytes in deep lakes have in general a hump‐shaped depth diversity gradient (DDG) for alpha richness, a prevailing decreasing pattern for beta richness and a dominantly hump‐shaped pattern for gamma richness (Figure 2a–c) (question 1.1). As we had only significant differences between mid and greater depths for all richness components, an even coarser species mapping resolution might be helpful. Our results are congruent to the few existing studies, which also show a hump‐shaped pattern (Ye et al., 2018) for alpha richness of submerged macrophytes. A simple explanation for the predominantly hump‐shaped pattern of alpha and gamma richness might be the mid‐domain effect (Colwell et al., 2004): Niches along environmental gradients overlap and build a peak of richness following geometric constraints. Furthermore, the generally decreasing beta DDG might be explained by a change in local species between transects in shallower depths. In shallow water, disturbances resulting from the surface might be more diverse, which may increase coexistence with spatial partitioning of occurrences. As disturbances are coming from the surface, we propose the hypothesis that shallow water has a higher environmental heterogeneity which might be the reason for an increased beta diversity.

We see a high variety of DDG shapes between lakes, as all these DDGs vary in their maximum richness (*R*_max_) and the corresponding depth (*D*_max_), but a robust hump‐shaped pattern can be seen for alpha richness (Figure 2d–f) (question 1.2). The high variety of *R*_max_ and *D*_max_ is not surprising, as we studied lakes showing a range of physical–chemical properties (Table 1) and gamma richness between 2 and 35 species per lake. This wide range of species richness and environmental conditions broadens our understanding of the DDG, previously limited to one single lake in China (Fu, Zhong, Yuan, Ni, et al., 2014; Fu, Zhong, Yuan, Xie, et al., 2014). Although for alpha and gamma richness, hump‐shaped curves along depth are predominant (Figure 2g–i), we also see increasing and decreasing patterns at single lakes. Increasing curves must be hump‐shaped, as we can safely assume that plant richness should decrease to zero further down in deep lakes. We detected more decreasing DDGs for gamma than for alpha richness, which reflects predominantly decreasing beta richness curves. Nevertheless, besides geometry and disturbances, there must be further variables affecting the DDG, as DDG shape varies between lakes, which themselves have different properties.

### 4.2 The DDG drivers

The drivers of the macrophyte DDGs strongly differed for DDG measures and richness components. Whereas pairwise correlations detected many strong relationships across richness components, multiple models revealed significant variables only for DDG measures of alpha richness.

The *R*_max_ correlate of the different richness components with a very similar set of abiotic parameters. All *R*_max_ correlate with *area*. This reflects species–area relationships (SAR) (Connor & McCoy, 1979; Lomolino, 2000; Patiño et al., 2014; Qian et al., 2007) for macrophytes, which is also shown by high correlation of *area* with total gamma richness. Looking at the GAMM results for nonlinear responses, *R*_(β,max)_ and *R*_(γ,max)_ did not show any significant results, but *R*_(α,max)_ is exclusively influenced by the *“SiO_2_ & Conductivity axis”* (PC1) (Figure 3). *Area, SAC*, and *WLF* also have a high contribution to PC1 and thus, *area* might be the key driving force again. The positive SAR of macrophytes was already shown in several studies (see Alahuhta et al., 2020 for a review). Moreover, a study comparing SAR of macrophytes with terrestrial plants would be very informative. For this purpose it would be interesting to add information about lake bathymetry, as lake *area* is just a proxy for the colonizable area per depth. Still, very generalizing indices like volume development index are also not suitable to determine the colonizable area in this case because the lake's morphology is very diverse (from kettles with several deep funnel‐shaped basins to v‐shaped glacial lakes and lake basins created by glacial erosion). However, it was not shown yet that the size of lakes also influences the shape of DDG.

The *D*_(β,max)_ and *D*_(γ,max)_ could not be explained with abiotic variables, neither by correlations nor by a GAMM. Unlike *D*_(α,max)_, the gamma, and consequently beta, values along DDG are more variable, indicating spatial heterogeneity and possibly unsaturation (Karger et al., 2014). Still, *D*_(α,max)_ correlates positively with *P_tot_* and *Tempsd* (higher *P_tot_* and *Tempsd* evoke a *D*_(α,max)_ in shallower water) and negatively with *O_2__diss_* and *Transp*. Furthermore, looking at nonlinear influences, the *D*_(α,max)_ is affected by all four PCA axes.

The PC2 (*Temperature & P_tot_ axis*) shows the highest influence (Figure 3). This means that in lakes with high *P_tot_* and/or high *Temp* the DDG peaks in shallower waters. Phosphorus is the limiting factor for phytoplankton growth and phytoplankton reduces the light availability for macrophytes. In contrast, it is still debatable whether the phosphorus concentration in the water is a limiting growth factor for macrophytes (Carr et al., 1997). One important point to consider in this debate is that rooted submerged macrophytes can also take up nutrients from the sediments (Lacoul & Freedman, 2006). Hence, phosphorus might affect macrophytes by promoting phytoplankton growth, which then reduces light availability and shifts DDG to shallower depths. Besides phosphorus, temperature is a major factor influencing metabolic processes as photosynthesis and respiration. Additionally in lakes, higher and prolonged high temperature result in higher nutrient levels due to increased mineralization and internal fertilization processes (Moss, 2012). Internal fertilization processes occur when longer high water temperatures lead to increased layering stability, prolonged oxygen consumption, anoxia in deep waters, resulting in anoxic resuspension of phosphorus from the lake sediments. These resuspended nutrients promote phytoplankton growth, thus reducing light for macrophytes. Therefore, the PC2 (*Temperature & P_tot_ axis*) describes the productivity gradient in lakes, caused by lower light availability leading to a shallower maximum of species richness.

Besides light quantity, light quality also influences *D*_(α,max)_, which is indicated by the influence of the PC4 (*O_2__diss_ – SAC axis*). With high *O_2__diss_* content and low *spectral absorption coefficient at 254nm* (*SAC*, a measure of colored dissolved organic matter*— CDOM*), we observe richness peaks at deeper waters. On the one hand, *CDOM* reduces damaging UV‐B radiation. On the other hand, it reduces light availability. Thus, we see a diametrically opposed effect of light quantity and light quality which might contribute to the prevailing pattern of highest species richness at medium depth level. In general, if light resource represents the main component of productivity in lakes, the mid‐depth DDG might follow the intermediate productivity hypothesis (Huston, 2014; Rajaniemi, 2003; VanderMeulen et al., 2001).

Besides light, *temperature* (*Temp*) also seems to influence *D*_(α,max)_, via surface water temperature and its influence on light availability (as explained above) and via the lake's layering depth. This second mechanism by which temperature layering affects DDG is demonstrated by the fact that along PC3 (*Tempsd—Chloride axis*) *D*_(α,max)_ decreases. A high *Tempsd* (shallow epilimnion—the upper temperature layer in a stratified lake) promotes a shallow *D*_(α,max),_ while a low *Tempsd* (broad epilimnion) allows deeper *D*_(α,max)_. *Tempsd* is positively correlated to *Temp* demonstrating that higher temperatures can lead to a shallower upper warm layer in water bodies as the stratification is more stable (Adrian et al., 2009).

The weakest influencing effect (lowest drop contribution) is provided by PC1 (*NH4^+^—SiO2 & Conductivity axis*). Just at very high values of PC1 *D*_(α,max)_ becomes shallower. As *Cond* is negatively correlated with *Transp* (cor = −0.71, *p* < .001), we speculate that also here *T* *ransp* is the actual mechanism that influences *D*_(α,max)_.

In summary, the main influences on *D*_(α,max)_ seem to be, as expected, factors of water quality that influence light quantity (*transparency*, influenced by *phosphorus* and *temperature*), light quality (*CDOM*), and layering depth (*temperature*). The main influence on *R*_(α,max)_ is the lake surface *area*.

### 4.3 DDG temporal change

We showed that the stability of the pattern depends on the DDG measure (question 3.1). *D*_(β,max)_ and *D*_(γ,max)_ were quite variable measures over years, while *D*_(α,max)_, *R*_(α,max)_, *R*_(β,max),_ and *R*_(γ,max)_ are comparatively stable measures. This may be related to the fact that there is neither pairwise correlation between nor an explaining model for *D*_(β,max)_ and *D*_(γ,max)_.

**FIGURE 4:**
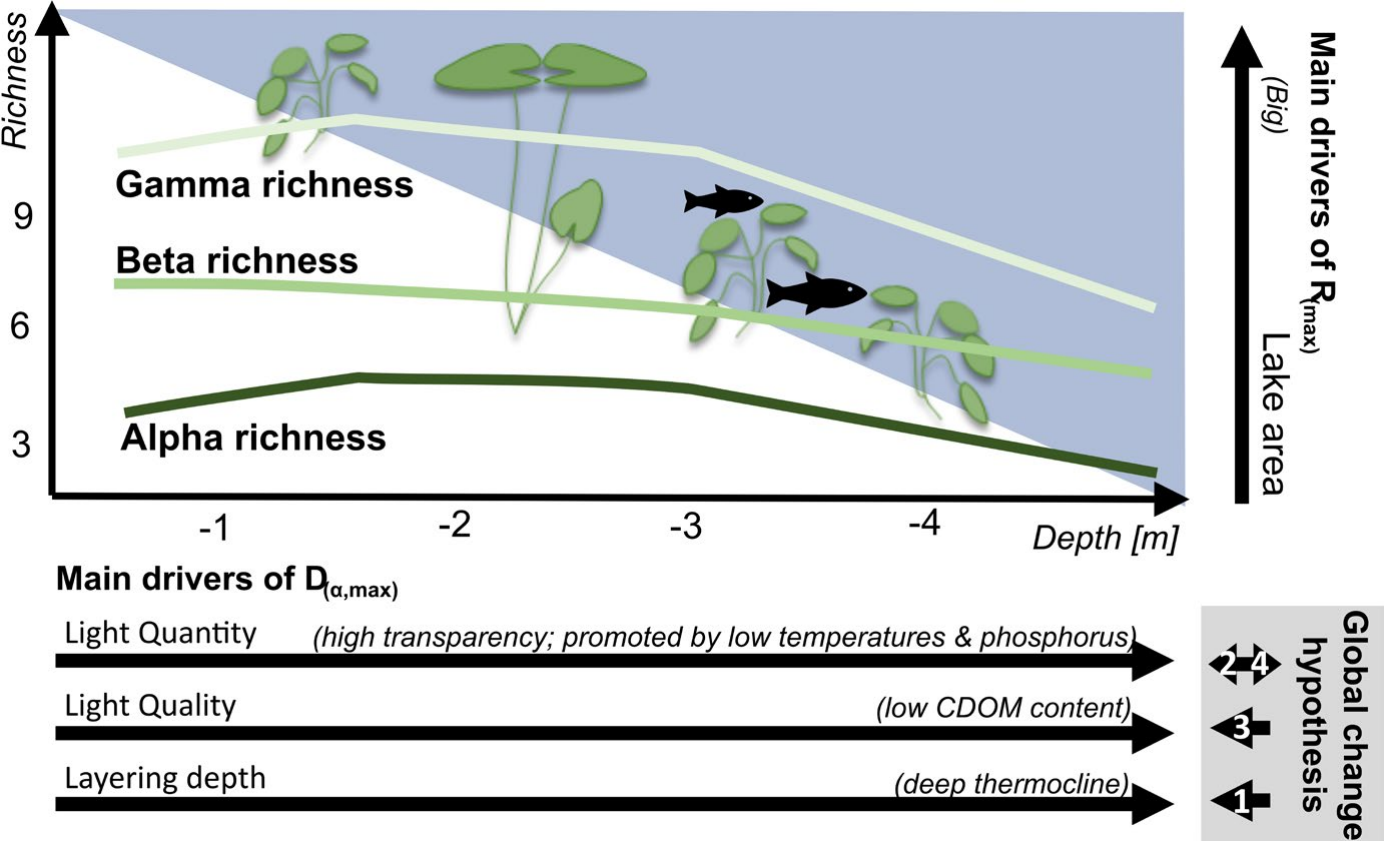
Summary figure showing the submerged macrophytes depth diversity gradient for alpha, beta, and gamma richness as well as the main drivers (black arrows) of the alpha richness peak, characterized by *R*_*(α,max)*_ and *D* *_(α,max)_*. Additionally, hypotheses for global change influences on the alpha richness peak for Bavaria are given in the gray box. The hypotheses are: (1) layering depth might become shallower due to rising water temperature. (2) Light quantity might be lowered due to lowered transparency. (3) Light quality is said to decrease. (4) Light quantity might be increased if water management gets adapted

Contrary to our expectations, we see no general trend of increasing species richness or decreasing *D*_max_ (question 3.2). Although we observe high variety in DDG temporal change between lakes, the DDG temporal change for single lakes, especially for *D*_(α,max)_, is low and develops into different directions for different lakes. Still, we see linear trends that are consistent over time within lakes. These patterns suggest that global change effects will be more complex than anticipated. In fact, climate and land use change influence all the highly connected chemical and physical gradients known to significantly affect DDG (Hossain et al., 2017). Thus, the following hypotheses can be formulated (Figure 4): (1) As temperatures rise, so do lake surface water temperatures (O'Reilly et al., 2015; Pilla et al., 2020). This seems to result in shallower epilimnion (Kraemer et al., 2015) and generally shallower *D*_max_ and a lower *R*_max_. (2) Furthermore, rising temperatures entail higher phosphorus content, as they promote internal fertilization. But extreme weather events combined with enriched fertilization in agriculture can also cause fertilization events (Rose et al., 2016), which might result in shallower light depth and consequently in shallower DDG. (3) Browning, which is generally increasing due to temperature‐induced decomposition rates and changes in precipitation events (Guarch‐Ribot & Butturini, 2016; Sobek et al., 2007; Weyhenmeyer & Karlsson, 2009), leads to a shallower *D*_max_. (4) However, water management reduced the external nutrient loading of European lakes enormously during the last decades (Eigemann et al., 2016; Murphy et al., 2018). This trend is still ongoing and might still lead toward lower nutrient contents and thus to deeper *D*_max_. All these opposing environmental trends make it hard to draw a general trend for multiple lakes for short timespans. However, for long timespans it seems to be a race between climate change impacts (Hypothesis 1–3 in Figure 4) that might lead to a shallower *D*_max_ and thus generally less macrophytes and water management impacts that might deepen the *D*_max_ via improved water quality (Hypothesis 4 in Figure 4). In summary, this study sets a good comparison for future studies once longer time series become available.

### 4.4 Implications for diversity gradients and hypotheses in general

Comparing different diversity gradients might provide deeper insights into mechanisms shaping species richness. Here, the DDG of macrophytes brings advantages compared with other gradients. The DDG assembles over shorter spatial scales than the latitudinal diversity gradient (LDG) or the elevational diversity gradient (EDG), which implies a lower importance of dispersal or connectivity and an easier replicability. For LDG, the options for replicates are restricted to two hemispheres (Pontarp et al., 2019), whereas EDG comparative studies require a high logistic sampling effort (Kessler et al., 2011; Nogués‐Bravo et al., 2008). Because of these advantages, other small‐scale diversity gradients may also be insightful. One example is the vertical diversity gradient (VDG) from forest floor to tree crowns, which involves sharp gradients of light intensity, temperature, and humidity. Therefore, we discuss below the potential explanatory hypotheses with all mentioned diversity gradients.

One explanation of the observed hump‐shaped DDG might be the intermediate productivity hypothesis (IPH). The IPH states that at low productivity level (deep waters with low light quantity and low temperature) only few specialized species survive, whereas at high productivity level (shallow waters) only few competitive species survive. Previous LDG study of freshwater plants revealed its peak at subtropical to low tropical latitudes (Murphy et al., 2019), thus peaking at intermediate level of solar productivity and reflecting our analysis of DDG. Intermediate light intensity and temperature would also match the mid‐canopy VDG peak for vascular epiphytes (Acebey et al., 2017; Krömer et al., 2007; Petter et al., 2021). Although quantification of productivity along depth should be attempted, our findings and the evidence from other diversity gradients already indicate a key role of light quantity and temperature in shaping DDG.

Another hypothesis is the mid‐domain effect (MDE), which proposes mid‐gradient peaks due to geometric constraints (Colwell et al., 2004). If depth ranges from shallow‐water species overlap with ranges of deep‐water species, a species richness peak in the middle of the gradient can be expected. The MDE is used to explain hump‐shaped patterns of the LDG (Pontarp et al., 2019) and the EDG (Colwell & Lees, 2000). Indeed, the overlap of light and temperature preferences may explain the subtropical peak (Murphy et al., 2019) in LDG of macrophytes and our reported mid‐depth peak in DDG as well as the mid‐canopy VDG peak in vascular epiphytes (Petter et al., 2021). Still, an adequate evaluation of the MDE requires quantification of environmental preferences for each species—an important direction for future empirical research. In this regard, the MDE evaluation may be more feasible to perform for DDG, as it considers a smaller regional species pool than the LDG and a better experimental feasibility than VDG given the faster life cycles of macrophytes compared with vascular epiphytes.

Another explanation is the intermediate disturbance hypothesis (IDH). It suggests species richness peaking at mid‐levels of disturbance as species of early and late successional phases coexist (Connell, 1978). Whereas the disturbances along EDG are caused by human activities at lower elevation (Nogués‐Bravo et al., 2008) and the disturbances along VDG can be associated with higher branchfall toward the outer crown of a tree (Cabral et al., 2015; Petter et al., 2021), depth‐dependent disturbances in water can be caused by anthropogenic use, waves, herbivory, ice cover, and water‐level fluctuations (Evtimova & Donohue, 2016). *Water‐level fluctuation* was already integrated in our study in a very simple way, but showed no strong effect on richness, thus did not explain the DDG. Nevertheless, considering that several disturbances in shallow waters should happen in lakes, future monitoring schemes should quantify more types of disturbances.

### 4.5 Limitations and perspectives

The main limitation is that, in some lakes, the deeper end of the DDG was not clearly quantified. This is, however, most critical for the lakes with increasing DDG (for alpha richness: Eibsee 2016; for beta richness: Eibsee 2011, Grosser Ostersee 2008 & 2014, Tachinger See 2006, Woerthsee 2008, Eibsee 2016, Schliersee 2008; for gamma richness: Eibsee 2011 & 2016, Grosser Ostersee 2008, Tachinger See 2006 and Woerthsee 2008). For these lakes, which are mostly lakes with a high water transparency, it might be interesting to split up the lowest depth level to have a finer resolved depth gradient and to quantify a metric termed “the lower macrophyte limit” (Søndergaard et al., 2013). This metric is often used as indicator for water quality and might be useful to further characterize the DDG as it defines the lower limit and the occupied space.

Additional limitations of our analyses can be viewed rather as perspectives for further studies focusing on explaining the underlying causes of the DDG (see previous section) and to disentangle the presented hypotheses, as these limitations require data yet unavailable. This includes (a) depth measurements of the variables that also show depth gradients (i.e., light, temperature, or nutrients) and (b) further variables that vary across transects and lakes, such as littoral area, transect distance, slope, soil properties (components, grain size distribution and nutrient content), average lake depth, ice cover duration, productivity, and different disturbance factors like anthropogenic use intensity (boats, mowing, swimming) or herbivory pressure (fish, water birds). Nevertheless, our analyses already indicate that light quality and quantity may play a main role in forming the DDG in freshwater lakes and will inspire further empirical studies on the DDG as well as comparative studies with other diversity gradients.

A promising direction for future research might be combining eco‐physiological experiments with mechanistic modeling to test the different species richness hypotheses. Such an approach might help to clarify the influencing force of disturbances or geometry on DDG on small scales.

## 5 CONCLUSION

Our study makes a step toward a cross‐lake generalizable understanding of the depth diversity gradient (DDG) of submerged macrophytes, their regional and temporal heterogeneity as well as the drivers of the DDG shape. Submerged macrophyte richness peaks predominantly at intermediate depth forming a hump‐shaped pattern for alpha and gamma richness, but a decreasing pattern for beta richness (Figure 4). Well‐known hypotheses of biogeography shape diversity gradients in general, such as mid‐domain effect and mean–productivity hypothesis. The latter is already supported by our findings on the role of light and temperature as DDG drivers. The key advantage of DDG in contrasting these hypotheses is the logistic feasibility of short‐distance scales and the exclusion of confounding effects associated with dispersal constraints. The key drivers of DDG we determined were area influencing the species richness peak height (*R*_(α,max)_) and light quality, light quantity, and layering depth influencing the species richness peak depth (*D*_(α,max)_). However, as there are many other possible factors for which we did not have data but which could play a role, further research is needed before general conclusions can be drawn from this study. Although we found that the DDG in general remained stable over the past few years for most lakes, we still found shifting trends for richness metrices for some lakes. However, these trends were shown to be diverse across lakes. Whereas climate change might be more ubiquitous, land use change may be lake‐specific. This suggests that water management strategies should also consider, besides global warming, lake characteristics, and change in the surrounding land use. The interaction of these aspects also means that although higher temperatures lead to a reduction in the quantity of light available to aquatic plants in lakes, land use measures can be taken to counteract this. Nevertheless, our findings already indicate that warmer water temperatures may still lead to a shift in species along depth towards shallower waters dependent on further efforts to hold or increase water quality of lakes.

## CONFLICT OF INTEREST

The authors declare that they have no conflict of interest.

## AUTHOR CONTRIBUTIONS

**Anne Lewerentz:** Conceptualization (equal); data curation (lead); formal analysis (lead); visualization (lead); writing—original draft (lead); writing—review and editing (lead). **Markus Hoffmann:** Writing—original draft (supporting); writing—review and editing (supporting). **Juliano Sarmento Cabral:** Conceptualization (equal); formal analysis (supporting); funding acquisition (lead); writing—original draft (supporting); and writing—review and editing (supporting).

## Supporting information

Supplementary Material

Supplementary Material

## Data Availability

Original raw data are publicly available, provided by Bayerisches Landesamt für Umwelt, www.lfu.bayern.de Processes data and code of data analysis are provided as Research Compendium: https://doi.org/10.5281/zenodo.5255571.
