# Supplementary material for: Depth diversity gradients of macrophytes: Shape, drivers, and recent shifts
Source: Ecol Evol. 2021 Sep 23;11(20):13830–45. doi: 10.1002/ece3.8089 (PMC8525089; doi:10.1002/ece3.8089)
Supplement: Supplementary file 1 — Supplementary Material [file ECE3-11-13830-s002.pdf]

Depth diversity gradients of macrophytes: shape, drivers and recent shifts

Supporting information I

Anne Lewerentz, Markus Hoffmann, Juliano Sarmento Cabral

Table of Contents

General information 2
Lake morphology 2
Depth diversity gradients (DDG) of macrophytes: shape 3
Correlations between richness components 3
DDG of all field campaigns 3
DDG per lake 4
Herberich test 7
DDG pattern types 7
Correlations between DDG metrices 8
Chi-square test of DDG pattern 8
Depth diversity gradients of macrophytes: drivers 9
Data representativeness of nested subsets 9
Correlations between drivers 10
Principle component analysis 12
GAMM for gamma richness & beta and gamma DDG measures 13
Alternative GAMM with parameters selected on expert knowledge 14
Depth diversity gradients of macrophytes: recent shifts 15
Invariability analysis 15
Temporal trend of gamma richness 15
General 15
Individual lakes 16
Temporal trend of DDG measures 17
General 17
Individual lakes 18
Literature 21

General information

Lake morphology

Table 1: Morphology of selected lakes: Lake surface area (*Area*) and depths of deepest point of the lake (*max. Depth*)

Lake	Area [ha]	Max. Depth [m]
**Chiemsee**	7990.00	73.4
**Starnberger See**	5636.20	127.8
**Ammersee**	4700.00	81.0
**Tegernsee**	893.40	72.2
**Staffelsee**	800.0	39.0
**Waginger See**	661.00	27.0
**Simssee**	649.00	22.5
**Kochelsee**	600.00	66.0
**Koenigssee**	500.00	190.0
**Woerthsee**	433.90	34.0
**Gr. Alpsee**	247.30	22.7
**Tachinger See**	236.00	16.5
**Bannwaldsee**	228.00	12.0
**Schliersee**	222.00	40.3
**Pilsensee**	194.57	17.1
**Hopfensee**	194.00	10.4
**Riegsee**	188.47	15.4
**Grosser Ostersee**	177.63	29.7
**Eibsee**	177.00	36.0
**Niedersonthofener See**	135.50	21.3
**Weissensee**	134.65	24.7
**Langbuergener See**	104.00	37.3
**Alpsee bei Schwangau**	88.00	62.0
**Hartsee**	86.64	39.1
**Abtsdorfer See**	84.04	20.0
**Pelhamer See**	77.90	21.3
**Obersee**	57.00	51.0

Depth diversity gradients (DDG) of macrophytes: shape

Correlations between richness components

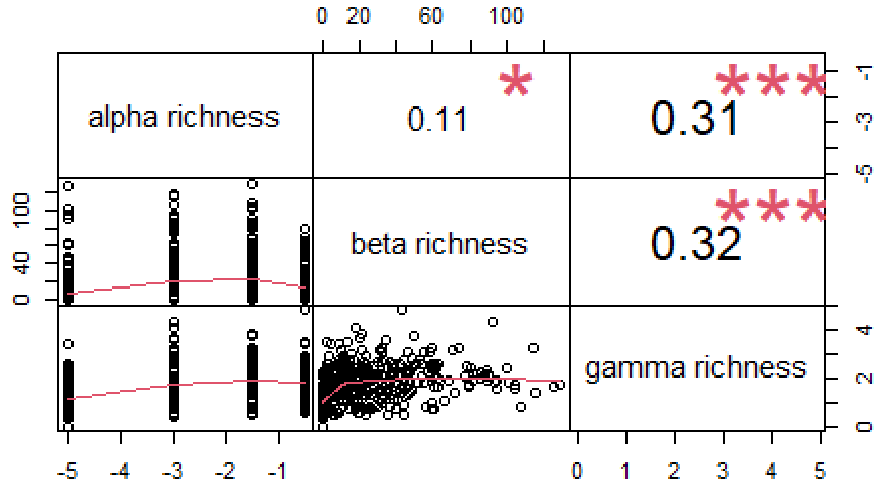

Figure 1: Correlations between diversity metrices (method = pearson). Significance levels of p-values: *p<0.1;**p<0.05;***p<0.01

DDG of all field campaigns

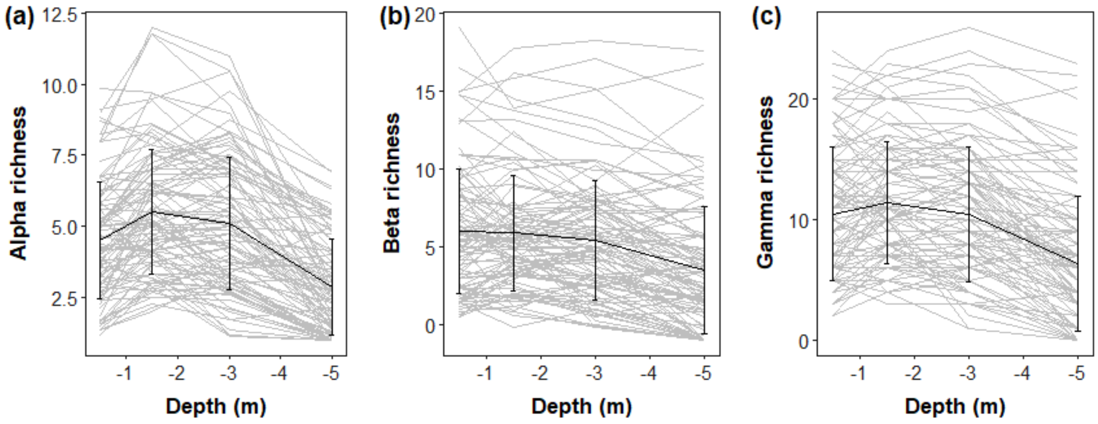

Figure 2: Depth diversity gradients of macrophytes for alpha (a), beta (b) and gamma richness (c) with mean as black line and sd as black bars. Each single grey line is one field campaign (lake*year).

DDG per lake

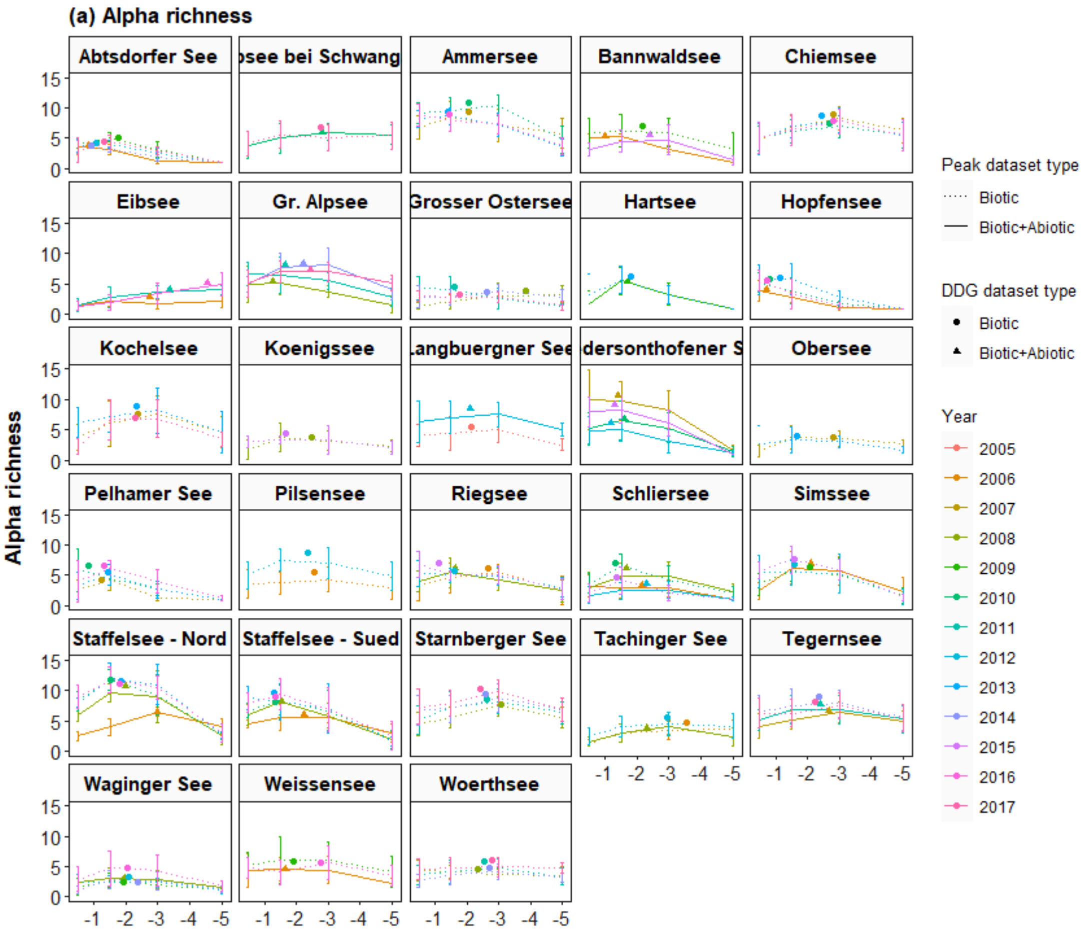

(a) Alpha richness
Abtsdorfer See
Ammersee
Bannwaldsee
Chiemsee
Eibsee
Gr. Alpsee
Grosser Ostersee
Hartsee
Hopfensee
Kochelsee
Koenigssee
Obersee
Pelhamer See
Pilsensee
Riegsee
Schliersee
Simssee
Staffelsee - Nord
Staffelsee - Sued
Starnberger See
Tachinger See
Tegernsee
Waginger See
Weissensee
Woerthsee
Alpha richness
Peak dataset type
Biotic
Biotic+Abiotic
DDG dataset type
Biotic
Biotic+Abiotic
Year
2005
2006
2007
2008
2009
2010
2011
2012
2013
2014
2015
2016
2017

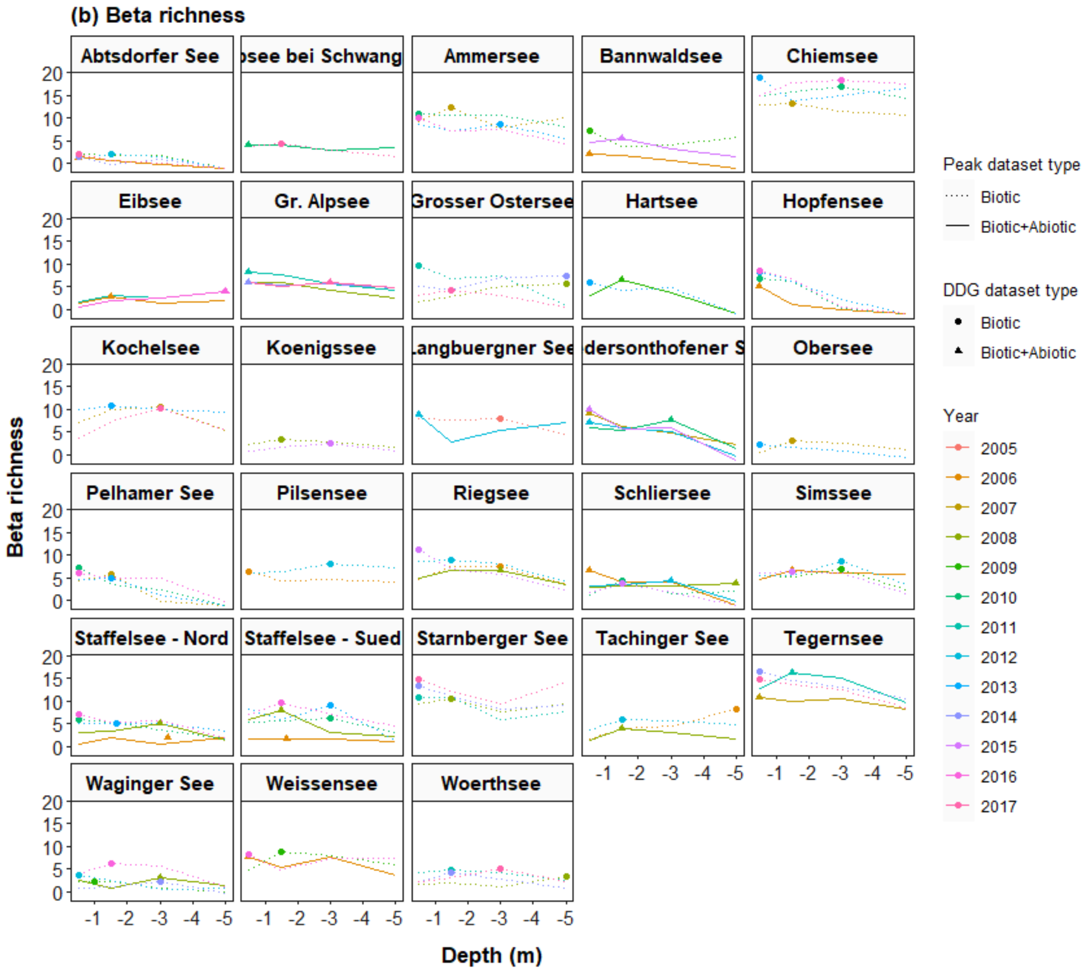

(b) Beta richness
Abtsdorfer See
psee bei Schwang
Ammersee
Bannwaldsee
Chiemsee
Eibsee
Gr. Alpsee
Grosser Ostersee
Hartsee
Hopfensee
Kochelsee
Koenigssee
Langbuergner See
dersonthofener S
Obersee
Pelhamer See
Pilsensee
Riegsee
Schliersee
Simssee
Staffelsee - Nord
Staffelsee - Sued
Starnberger See
Tachinger See
Tegernsee
Waginger See
Weissensee
Woerthsee
Beta richness
Depth (m)
Peak dataset type
Biotic
Biotic+Abiotic
DDG dataset type
Biotic
Biotic+Abiotic
Year
2005
2006
2007
2008
2009
2010
2011
2012
2013
2014
2015
2016
2017

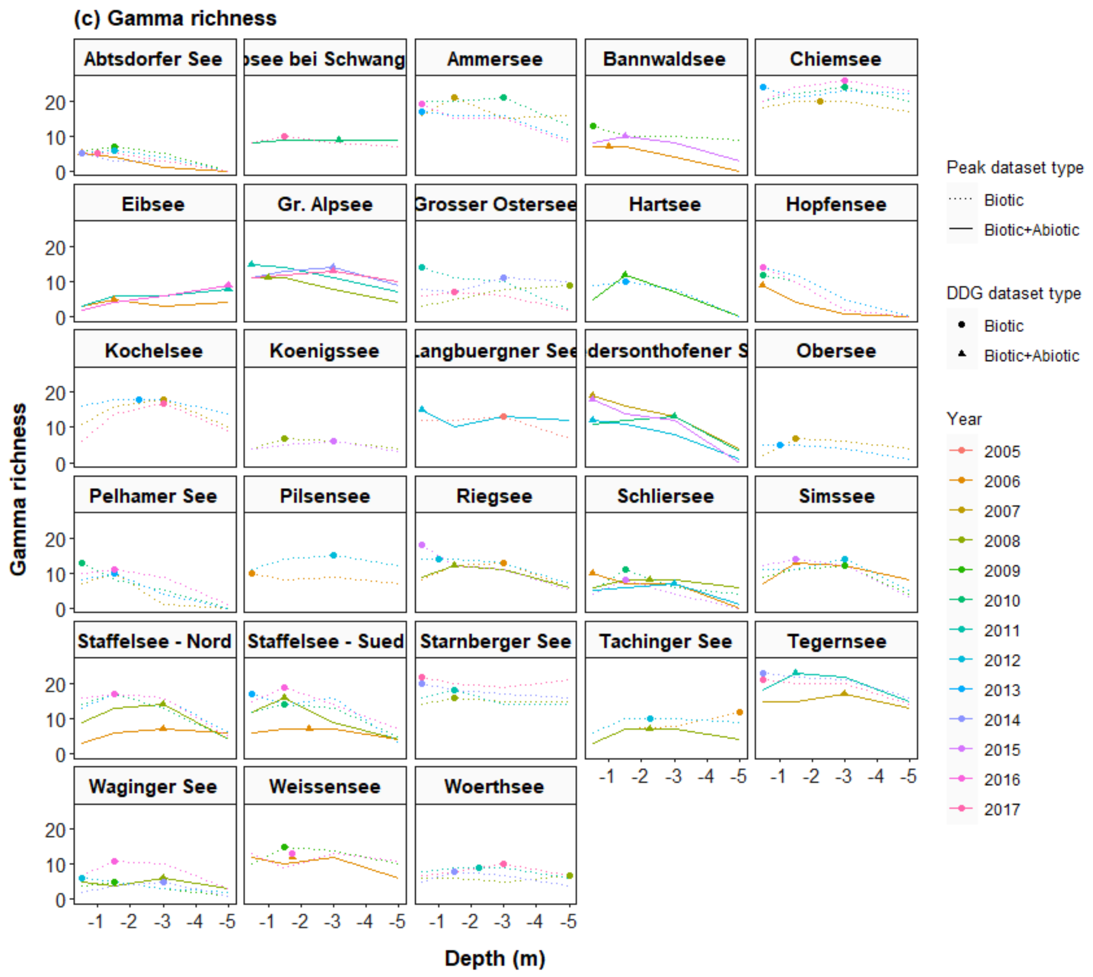

Figure 3: DDG of submerged macrophytes for alpha (a), beta (b) and gamma richness (c). For alpha richness, lines show the mean alpha richness per lake and year with their corresponding standard deviation; the single richness peaks (=DGG measures) are depicted as points. The different dataset levels can be distinguished by line type and point shape. Points and dashed line: Biotic dataset of all available macrophyte mapping (biodiversity dataset); triangles and solid line: subset of biotic dataset, where also abiotic data is available (environmental & biodiversity dataset).

Herberich test

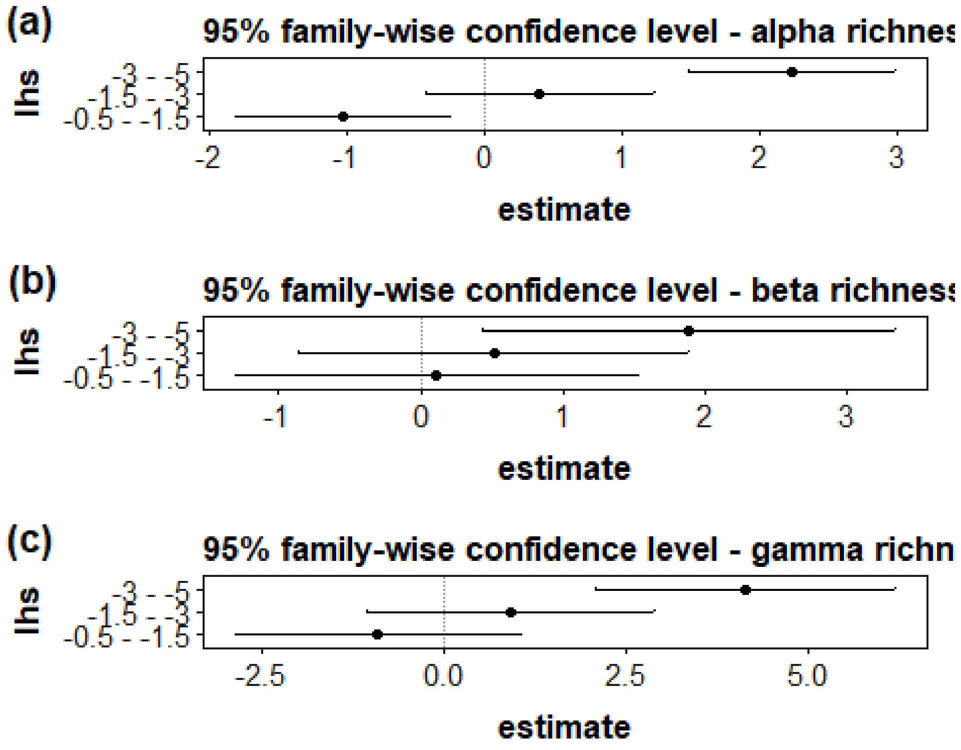

Figure 4: Simultaneous tests for linear models with multiple comparisons of means using Tukey contrasts that are robust under non-normality, heteroscedasticity and variable sample size (Herberich et al. 2010) to check significant differences between depths within richness components. Results are plotted for alpha richness (a), beta richness (b) and gamma richness(c).

DDG pattern types

Table 2: Overview about DDG patterns of decreasing curves, hump-shaped curves (different peak depths) and increasing curves. Number of field campaigns (lake*year) showing a distinct depth pattern for each richness component.

	**Decreasing curve (Peak: >-1m)**	**Hump-shaped (Peak: -1- -2m)**	**Hump-shaped (Peak: -2- -4m)**	**Increasing curve (Peak: <-4m)**
**Alpha richness**	6	41	52	1
**Beta richness**	40	31	22	7
**Gamma richness**	26	39	30	5

Correlations between DDG metrices

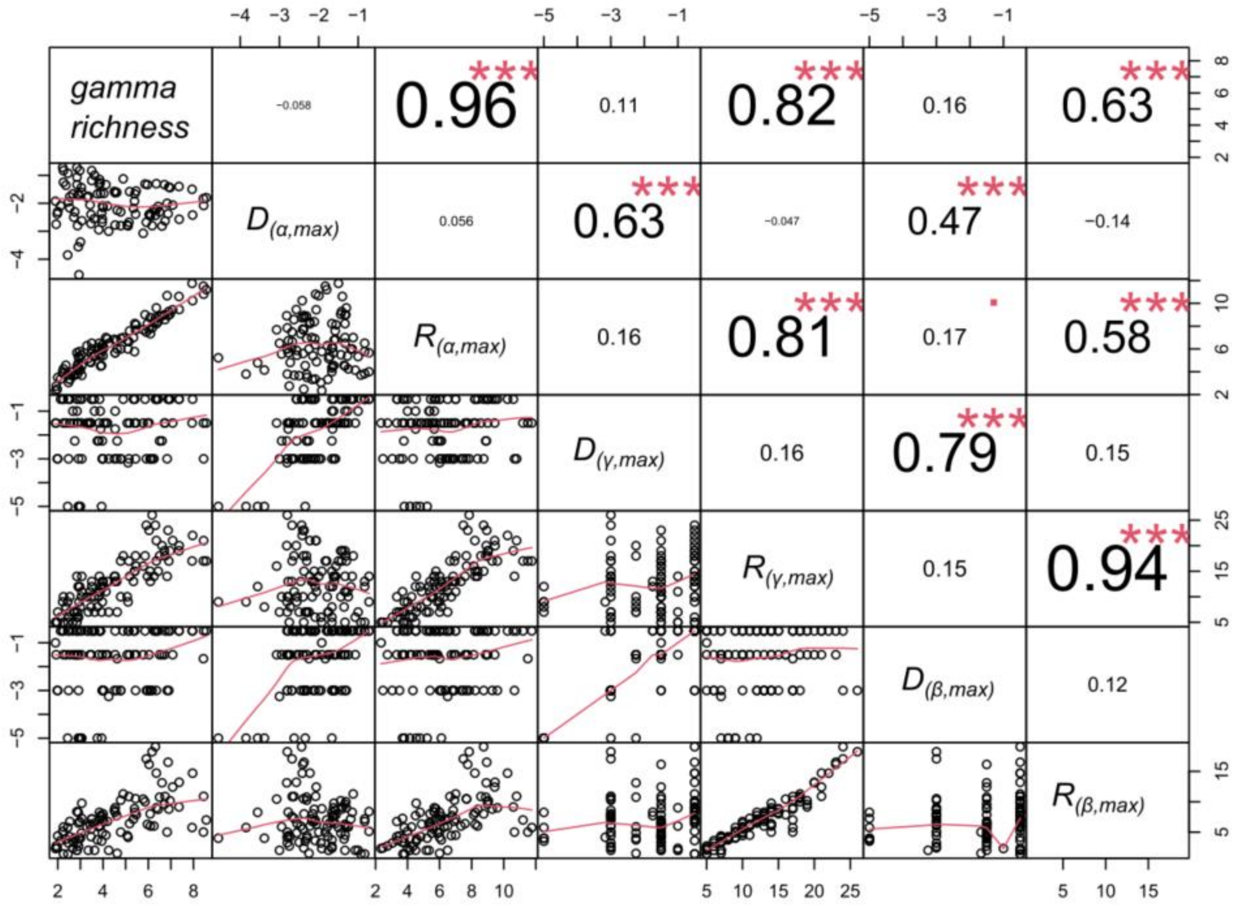

Figure 5: Pearson correlations between DDG measures of different species richness components. Significance levels of p-values: *p<0.1;**p<0.05;***p<0.01

Chi-square test of DDG pattern

Chi-square is done to see significant differences in frequency among the DDG pattern types (Table 2) to see if they are significantly different for each richness component.

```
##
##  Pearson's Chi-squared test with simulated p-value (based on 2000
##  replicates)
##
## data:  PEAKCLASS
## X-squared = 44.078, df = NA, p-value = 0.0004998
```

Depth diversity gradients of macrophytes: drivers

Data representativeness of nested subsets

To show that the diversity metrics of the environmental & biodiversity dataset are representative for the diversity metrics of biodiversity dataset we applied the PERMANOVA test adonis2, using the R package ‘vegan’ which compares centroids and the variance (Oksanen et al. 2019). A non-significant result ($p>0.05$) confirms that centroids and variance of two groups are not different. The results show that the *Environmental & biodiversity dataset* (N=27) is representative for the *Biodiversity dataset* (N=100).

```
## Permutation test for adonis under reduced model
## Permutation: free
## Number of permutations: 999
##
## adonis2(formula = PEAK[, c(3, 5)] ~ datasettotsimpl, data = PEAK, by =
NULL)
##          Df SumOfSqs       R2       F Pr(>F)
## Model     1  -25.160 -0.36115 -26.002  0.746
## Residual 98   94.827  1.36115
## Total    99   69.667  1.00000
```

```
## Permutation test for adonis under reduced model
## Permutation: free
## Number of permutations: 999
##
## adonis2(formula = (PEAK[, c(14, 15)]) ~ datasettotsimpl, data = PEAK, by =
NULL)
##          Df SumOfSqs      R2      F Pr(>F)
## Model     1   0.1690 0.02018 2.0189  0.128
## Residual 98   8.2047 0.97982
## Total    99   8.3738 1.00000
```

```
## Permutation test for adonis under reduced model
## Permutation: free
## Number of permutations: 999
##
## adonis2(formula = scale(PEAK[, c(16, 17)]) ~ datasettotsimpl, data = PEAK,
by = NULL)
##          Df SumOfSqs       R2       F Pr(>F)
## Model     1   -45388 -0.26182 -20.335  0.649
## Residual 98   218744  1.26182
## Total    99   173355  1.00000
```

Correlations between drivers

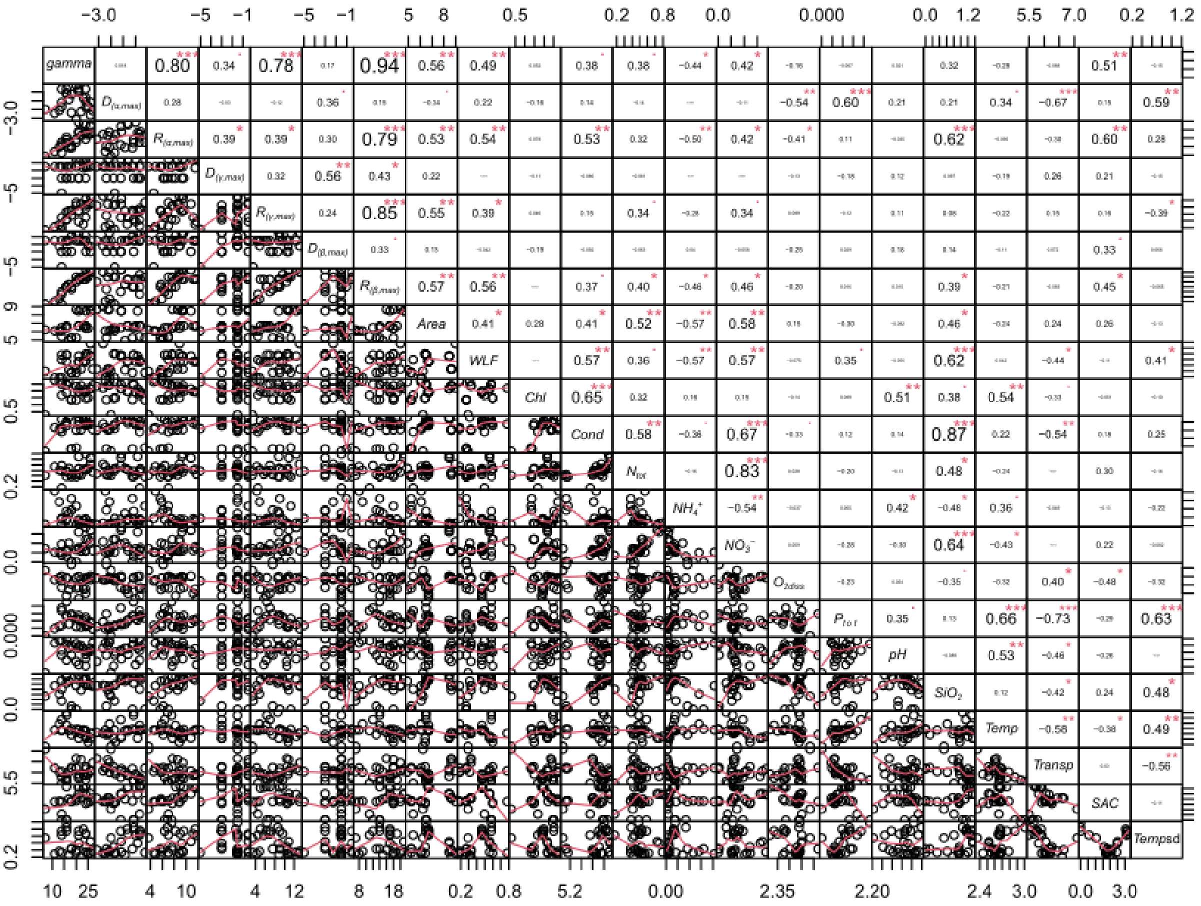

Figure 6: Pearson correlation between normalized chemical-physical values & DDG measures for all richness components. Environmental & biodiversity dataset is used. Significance levels of p-values: *p<0.1;**p<0.05;***p<0.01

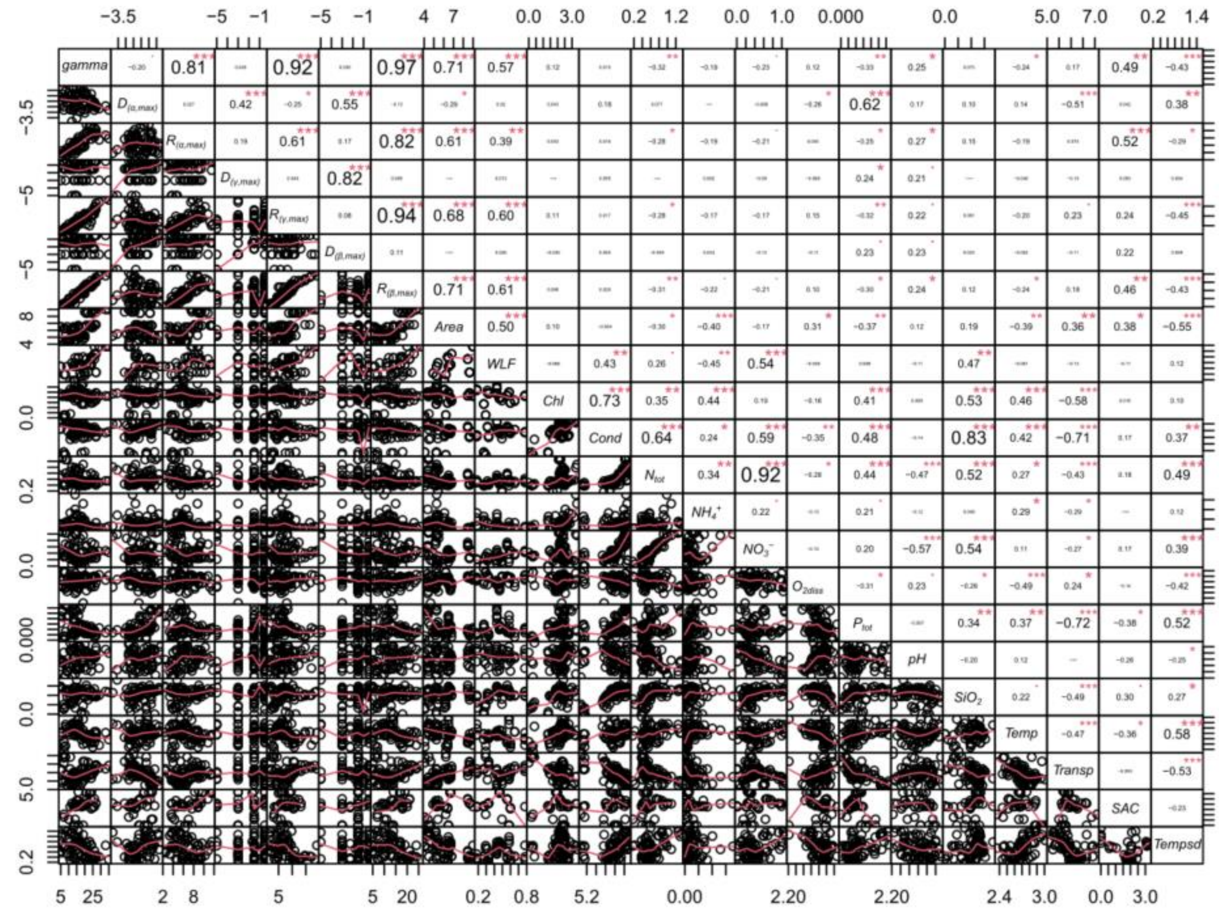

Figure 7: Pearson correlation between normalized chemical-physical values & DDG measures for all richness components. Biodiversity dataset is used, zero values are ignored. Significance levels of p-values: *p<0.1;**p<0.05;***p<0.01

Principle component analysis

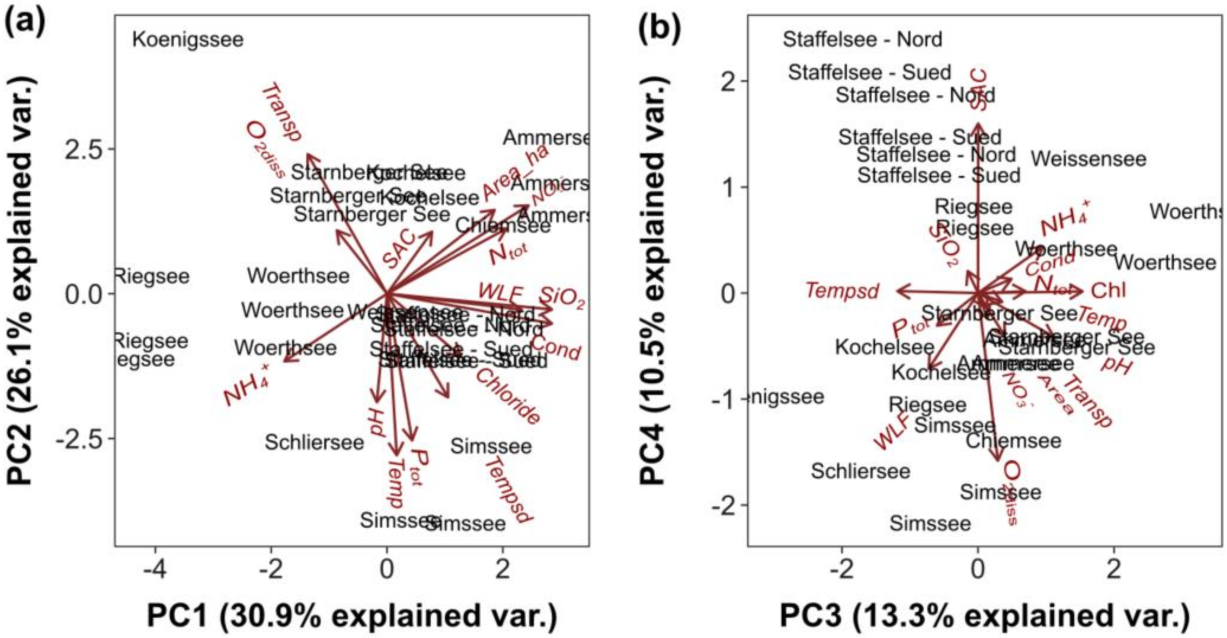

Figure 8: PCA with all environmental parameters of the environmental & biodiversity dataset. PCA axes 1-4 cover 0.8086% of variation. Axes 1 & 2 are plotted in panel (a), axes 3 & 4 in panel (b).

GAMM for gamma richness & beta and gamma DDG measures

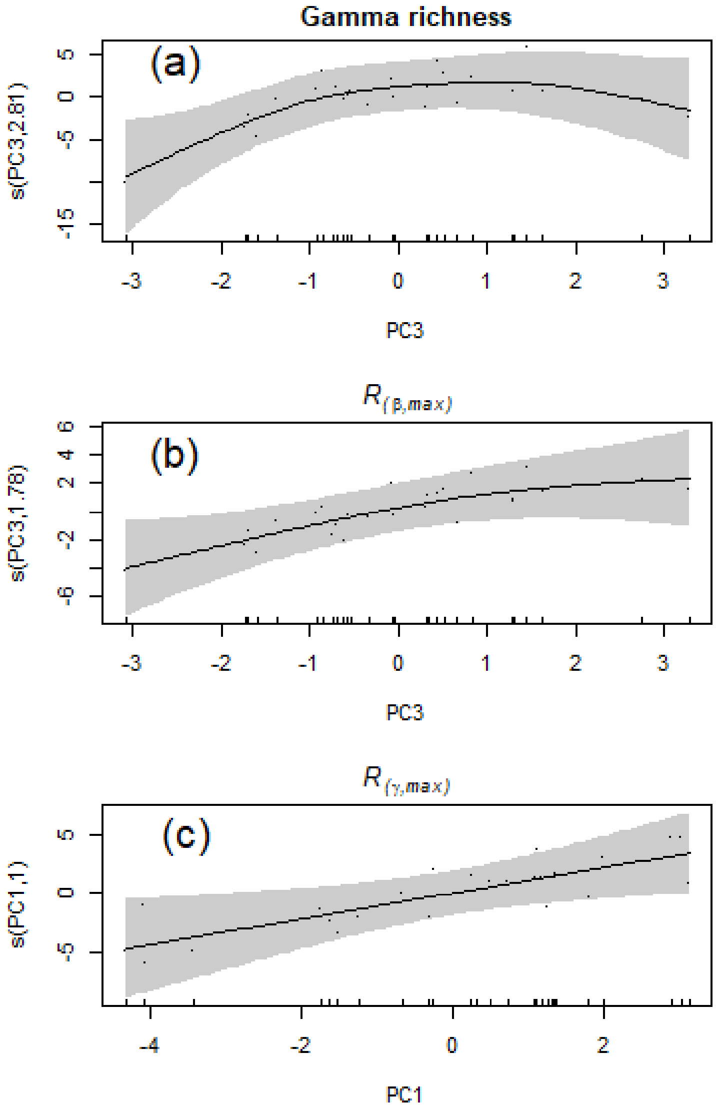

Figure 9: Minimal GAMMs for response variables Gamma richness (a), $R_{(\beta,max)}$ (b) and $R_{(\gamma,\, max)}$ (c). Low r-square (adj) were found: 0.213 (a); 0.0124 (b); 0.265 (c).

Alternative GAMM with parameters selected on expert knowledge

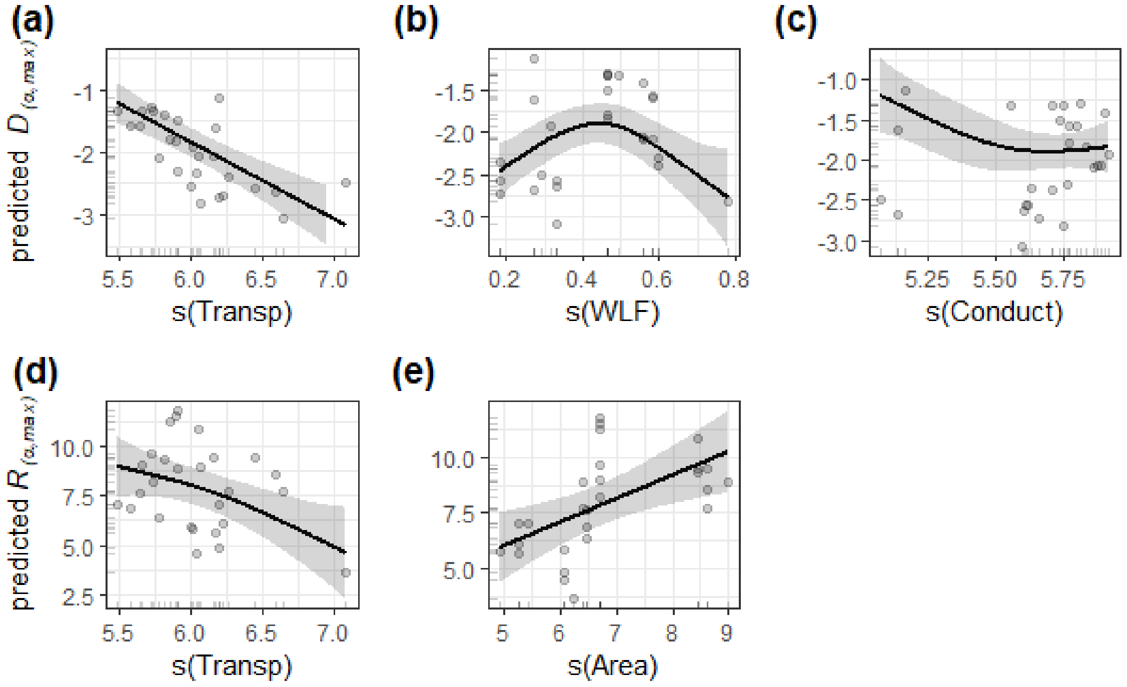

Figure 10: Alternative GAMMs without PCA for selected parameters. The selection was based on expert knowledge on highly likely influencing factors and excluding high correlations (see Figure 6 and 7). We selected *Cond* (representative for *chl*), N_{tot} (representative for NH_4^+ and NO_3^-), P_{tot}, *Temp* (representative for *Tempsd*), *Transp* (representative for *SAC*), *area* and *WLF*. We excluded *pH*, O_{2diss} & SiO_2. The resulting GAMM for $D_{(\alpha,max)}$ 0.697 (a-c) has a R^2 lower (R^2=0.697) than in the analysis using the PCA axes as variables (R^2=0.73). GAMM for $R_{(\alpha,max)}$ (d-f) shows also a slightly lower R^2 (R^2=0.432) than in the analysis using the PCA axes as variables (R^2=0.44).

Depth diversity gradients of macrophytes: recent shifts

Invariability analysis

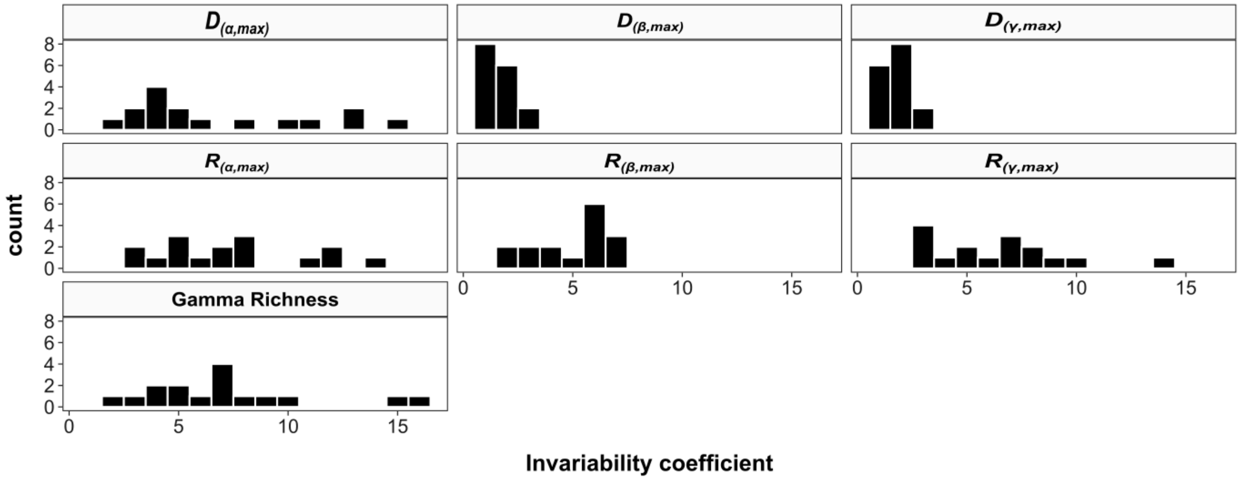

Figure 11: Histograms for Invariability coefficients for single DDG measures (D_{max} and R_{max} of alpha, beta and gamma richness) and gamma richness for the biodiversity timeseries dataset. High invariability means a high stability within the timeseries.

Temporal trend of gamma richness

General

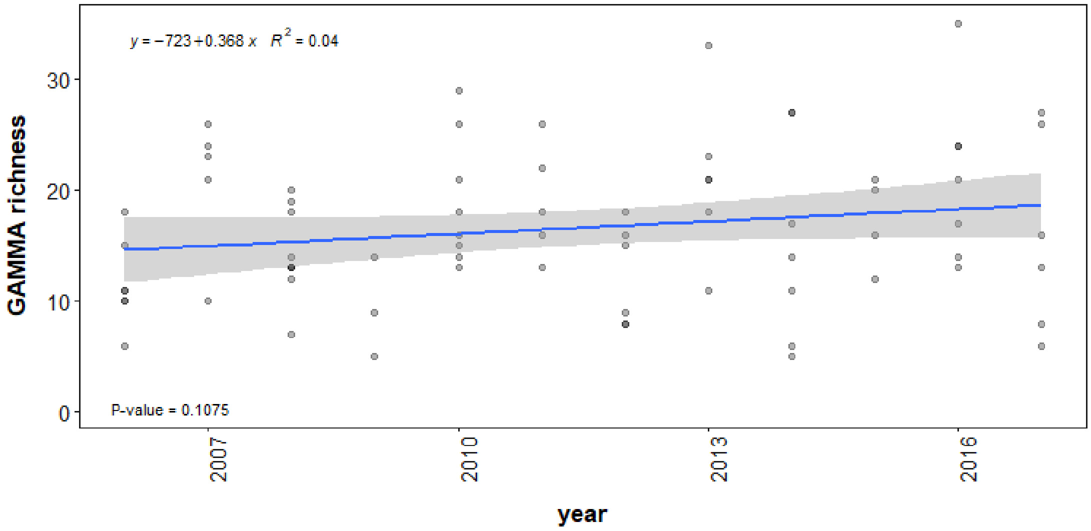

Figure 12: Temporal change of gamma richness from biodiversity time series dataset for all lakes together. Points show individual values, and the blue line is a linear model.

Individual lakes

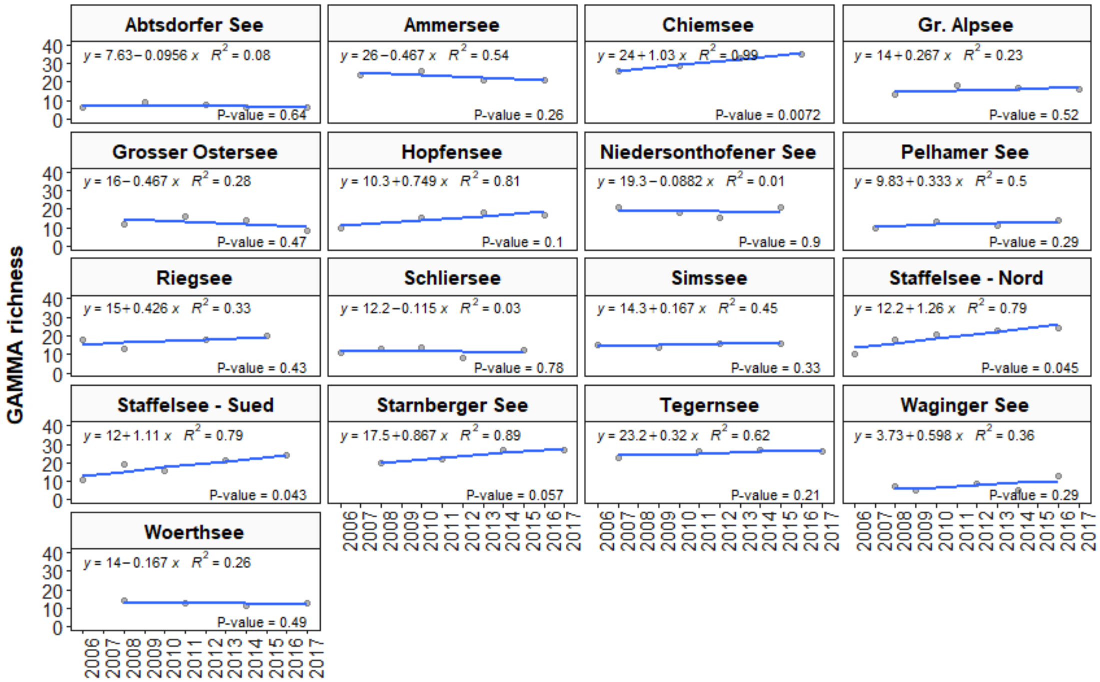

Figure 13: Temporal change of gamma richness from biodiversity time series dataset for all individual lakes. Points show single values, and the blue line is a linear model per lake.

Temporal trend of DDG measures

General

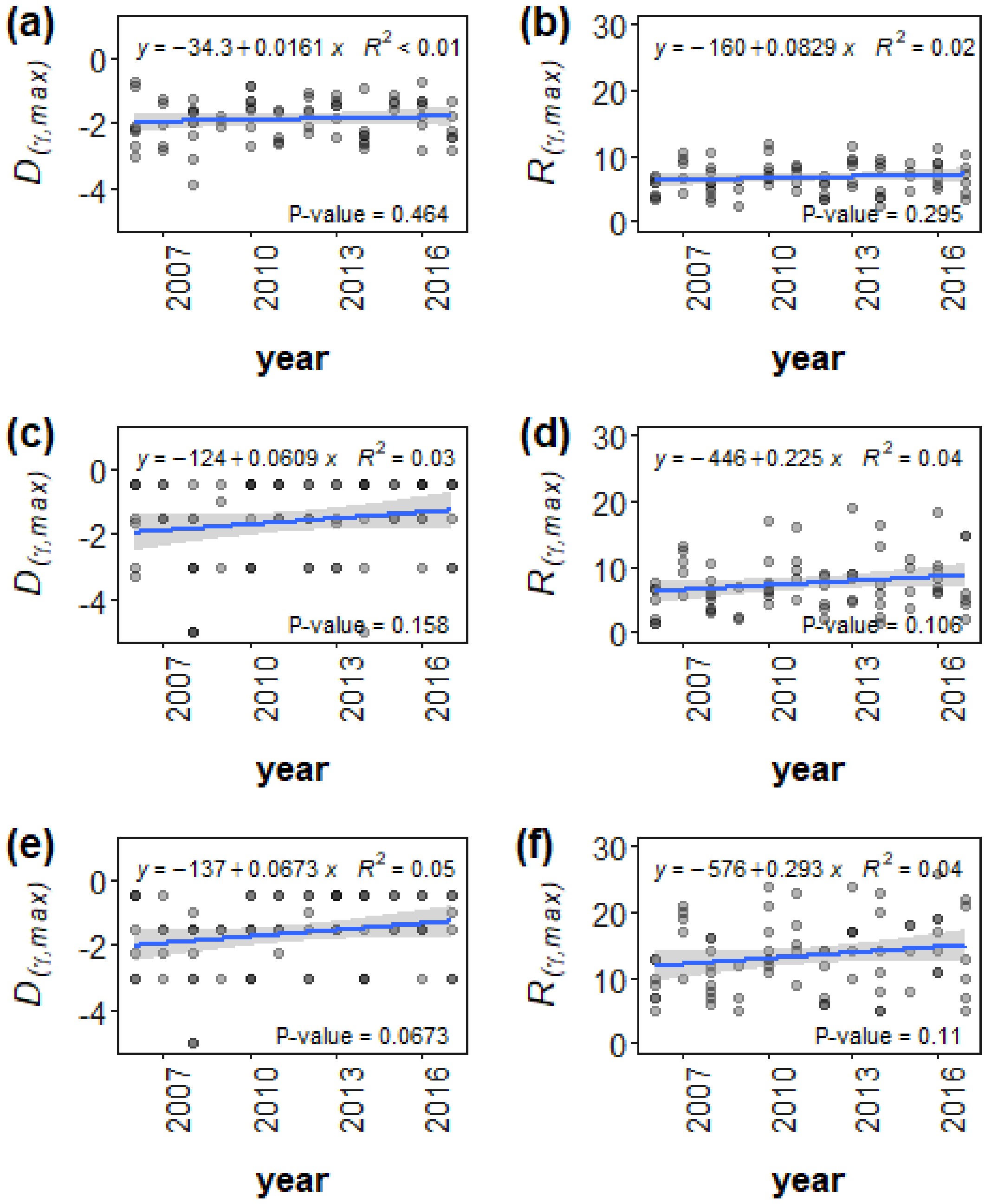

Figure 14: Temporal change of DDG metrices from biodiversity time series dataset for all lakes at once. Points show single values, and the blue line is a linear model. In panel (a,c,e) temporal change of $D_{(max)}$ is shown and in panel (b,d,f) the corresponding course of $R_{(max)}$ is depicted.

Individual lakes

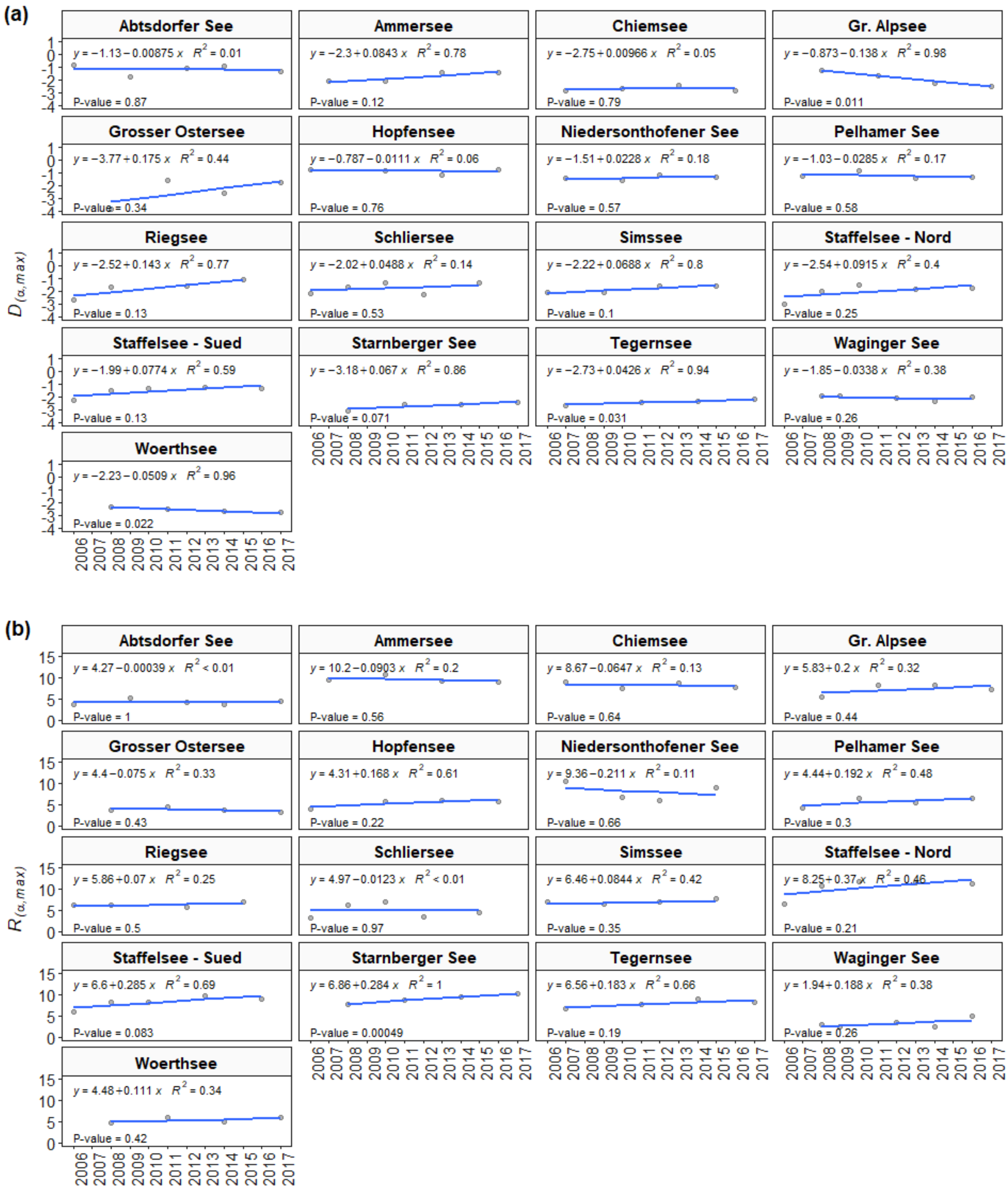

Figure 15: Temporal change of DDG metrices from biodiversity time series dataset for all lakes. Points show annual values, and the blue line is a linear model. In panel (a) temporal change of $D_{(\alpha,max)}$ is shown for all lakes and in panel (b) the corresponding course of $R_{(\alpha,max)}$ is depicted.

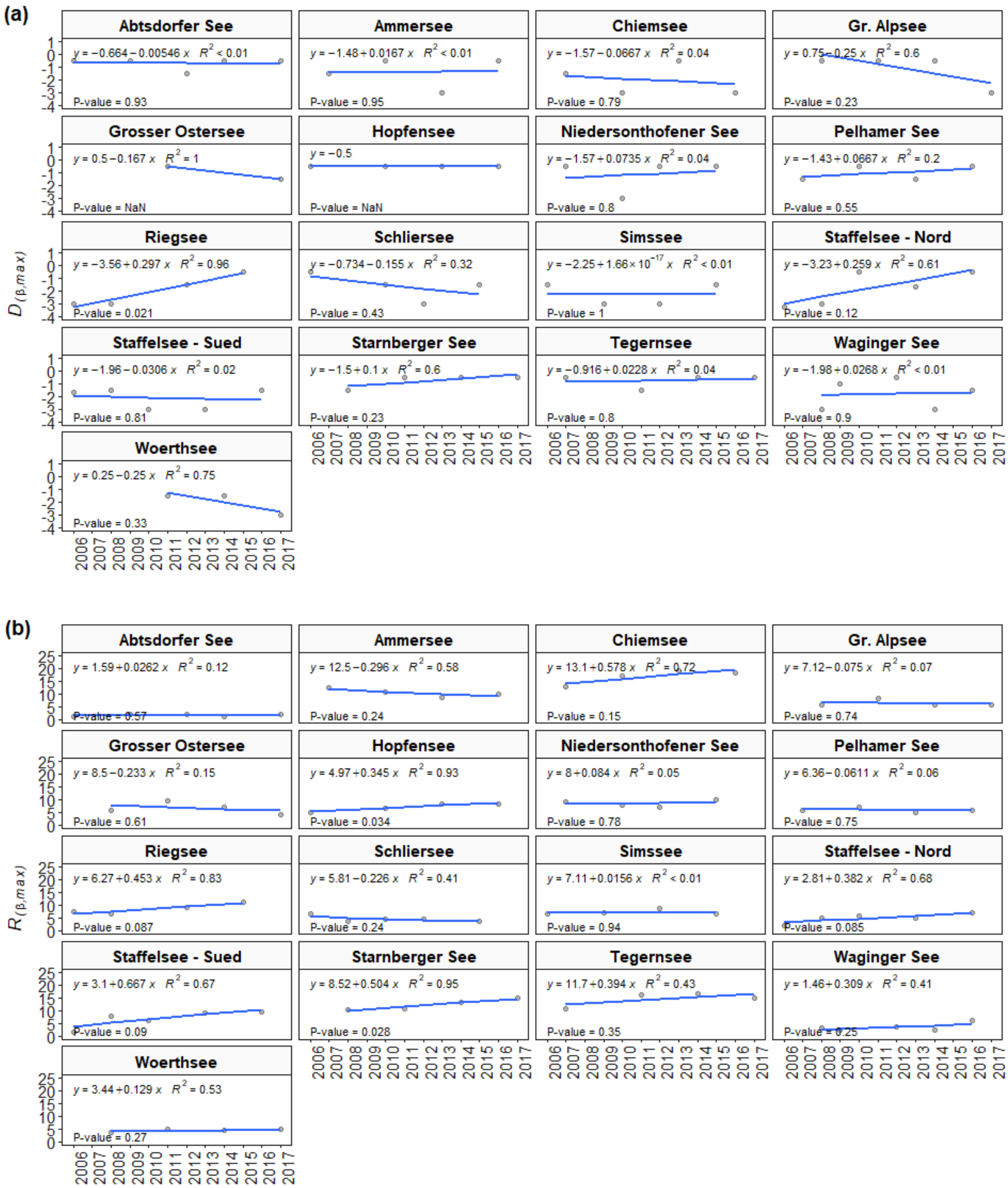

Figure 16: Temporal change of DDG metrices from biodiversity time series dataset for all lakes. Points show annual values, and the blue line is a linear model. In panel (a) temporal change of $D_{(\beta,max)}$ is shown for all lakes and in panel (b) the corresponding course of $R_{(\beta,max)}$ is depicted.

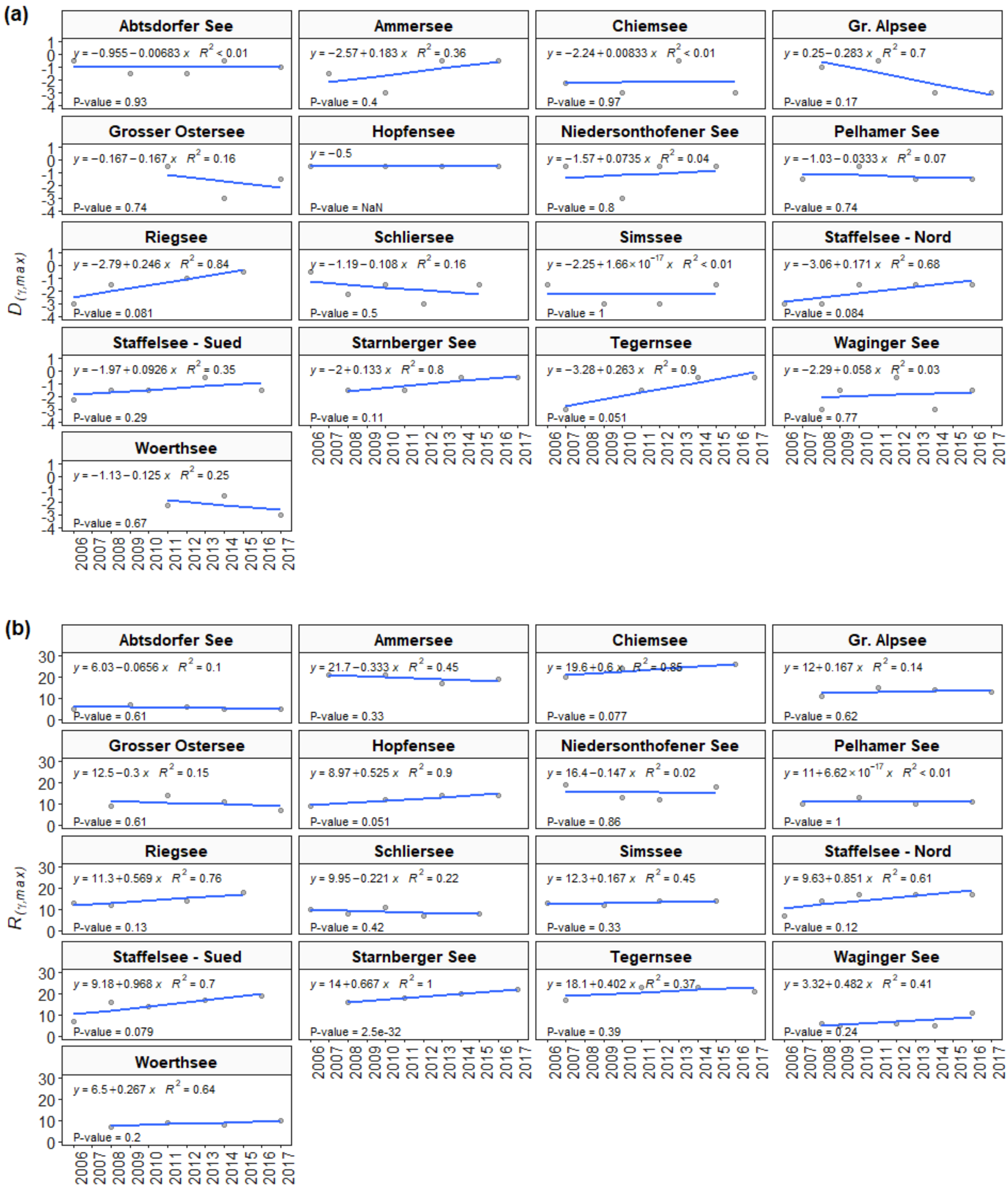

Figure 17: Temporal change of DDG metrices from biodiversity time series dataset for all lakes. Points show annual values, and the blue line is a linear model. In panel (a) temporal change of $D_{(\gamma,max)}$ is shown for all lakes and in panel (b) the corresponding course of $R_{(\gamma,max)}$ is depicted.

Literature

Herberich, E. et al. 2010. A Robust Procedure for Comparing Multiple Means under Heteroscedasticity in Unbalanced Designs (F Rapallo, Ed.). - PLoS ONE 5: e9788.
