# Supplementary material for: Depth diversity gradients of macrophytes: Shape, drivers, and recent shifts
Source: Ecol Evol. 2021 Sep 23;11(20):13830–45. doi: 10.1002/ece3.8089 (PMC8525089; doi:10.1002/ece3.8089)
Supplement: Supplementary file 2 — Supplementary Material [file ECE3-11-13830-s001.pdf]

Depth diversity gradients of macrophytes: shape, drivers and recent shifts

SECTION	QUESTIONS	HYPOTHESIS	DATASET	ANALYSIS	*FIGURES/TABLES*	RESULT
MATERIALS & METHODS					*Tab 1: Abiotic data; Fig 1: Experimental design*	
RESULTS	General overview		*biodiversity dataset (bd)*	0.1 Number of species per taxonomic groups;<br>0.2 Number of species per dataset;<br>0.3 Depth-indep. gamma richness range, mean, sd		(20 sp), Bryophytes (5 sp) and Pteridophytes (2 sp)<br>0.2 bd: 75 sp - e&bd: 57 sp - btd: 66 sp<br>0.3: 5-34; 15.36; 6.27
	1.1. What is the general shape of the depth diversity gradient (**DDG**) of submerged macrophytes in deep lakes?	Hump-shaped (as other study says)	*biodiversity dataset*	1. Plotting general DDG curves: mean and sd of Alpha, Beta and Gamma richness over depth<br>2. Herberich test: richness measures across depth<br>3. Classification in pattern types & histogram	*Fig 2: DDG, Peaks, Pattern type frequency for alpha, beta and gamma richness*	Alpha: prevailing hump-shaped pattern<br>Beta: prevailing decreasing pattern<br>Gamma: mainly hump-shaped, but not very clear<br>Herberich: only significant differences between mid and higher depths
	1.2. Are there differences between lakes and diversity components (alpha, beta, gamma richness)?	no broad differences between lakes and diversity components as the pattern is supposed to be generalisable	*biodiversity dataset*	4. Plotting single DDG peaks + regression line<br>5. Cor.test between richness measures<br>6. Cor.test between Peak measures across diversity components<br>7. Chi-square test: pattern types and richness components		Between lakes: Yes, there are differences. As all pattern types are represented for each richness component<br>DDG measures for all three richness components: hump-shaped pattern for alpha richness and a bi-modal pattern for beta and gamma richness.<br>Between div components: high correlations of measures across components,
	2. What are the **drivers** for macrophyte DDG?	high influencing force of water quality since water quality influence the available resources (light, temperature).	*environm. & biodiversity dataset (e&bd)*	8. Correlations within Abiotic variables & Richness measures<br>9. PCA of abiotic variables to reduce complexity<br>10. GAMM analysis	*Fig3: PCA loadings & GAMM results*	Correlations: $R(\alpha,max)$ - area, WLF, Conduct, NH4, SiO2; $R(\beta,max)$ - area; $R(\gamma,max)$ - area, WLF; $D(\alpha,max)$ - O2, Ptot, Transp, Tempsd; $D(\beta,max)$ & $D(\gamma,max)$ - none<br>GAMM: just significant models for $D(\alpha,max)$ & $R(\alpha,max)$; $D(\alpha,max)$: PC2, PC4, PC3, PC1<br>$R(\alpha,max)$: PC1
	3.1. Has the DDG being stable over recent years?	DDG is a quite stable pattern over time as macrophytes react slowly to changes	*timeseries dataset (btd)*	11. Inverse of coefficient of variation per lake over time		DDG is quite stable over years, especially for DDG measures of alpha richness
	3.2. Are temporal trends general or lake-specific?	General trend towards more species as water temp. increases and species invade.	*timeseries dataset*	12. Linear models: time series analysis of DDG variables (Dmax and Rmax) across richness components for each lake.	*Tab 2: Linear models for single lakes*	DDG shows linear trends of diversity metrices for single lakes, not for all lakes together; Gamma richness slightly trends toward more species.
DISCUSSION					*Fig. 4: Summary figure*	
